# Proton irradiation impacts age-driven modulations of cancer progression influenced by immune system transcriptome modifications from splenic tissue

**DOI:** 10.1093/jrr/rrv043

**Published:** 2015-08-07

**Authors:** Justin Wage, Lili Ma, Michael Peluso, Clare Lamont, Andrew M. Evens, Philip Hahnfeldt, Lynn Hlatky, Afshin Beheshti

**Affiliations:** 1 Center of Cancer Systems Biology, Tufts University School of Medicine, Boston, MA, USA; 2 Molecular Oncology Research Institute, Tufts Medical Center, Tufts Cancer Center, Tufts University School of Medicine, Boston, MA, USA

**Keywords:** aging and cancer, protons, spleen, tumor progression, tumor microenvironment, bioinformatics, immunosuppression, transcriptome analysis

## Abstract

Age plays a crucial role in the interplay between tumor and host, with additional impact due to irradiation. Proton irradiation of tumors induces biological modulations including inhibition of angiogenic and immune factors critical to ‘hallmark’ processes impacting tumor development. Proton irradiation has also provided promising results for proton therapy in cancer due to targeting advantages. Additionally, protons may contribute to the carcinogenesis risk from space travel (due to the high proportion of high-energy protons in space radiation). Through a systems biology approach, we investigated how host tissue (i.e. splenic tissue) of tumor-bearing mice was altered with age, with or without whole-body proton exposure. Transcriptome analysis was performed on splenic tissue from adolescent (68-day) versus old (736-day) C57BL/6 male mice injected with Lewis lung carcinoma cells with or without three fractionations of 0.5 Gy (1-GeV) proton irradiation. Global transcriptome analysis indicated that proton irradiation of adolescent hosts caused significant signaling changes within splenic tissues that support carcinogenesis within the mice, as compared with older subjects. Increases in cell cycling and immunosuppression in irradiated adolescent hosts with *CDK2*, *MCM7*, *CD74* and *RUVBL2* indicated these were the key genes involved in the regulatory changes in the host environment response (i.e. the spleen). Collectively, these results suggest that a significant biological component of proton irradiation is modulated by host age through promotion of carcinogenesis in adolescence and resistance to immunosuppression, carcinogenesis and genetic perturbation associated with advancing age.

## INTRODUCTION

Proton radiotherapy is a rapidly growing field of cancer treatment, due to greater normal tissue sparing and improved tumor treatment over standard radiotherapy in some cases. Since the majority of the energy is focused at the Bragg peak, the end of the track, higher dose of proton irradiation can be deposited within the tumor area [1]. Pre-clinical studies have shown that proton irradiation has unexpected biological advantages for tumor therapy, including anti-angiogenesis and impaired cell invasiveness [2, 3]. Determining unexpected translational effects of exposed non-tumor tissue to proton irradiation can also alter the dynamics between the tumor and the host, impacting therapeutics and carcinogenesis [1, 4]. For example, a reduction in both the size of lymphoid organs (i.e. spleen) and number of factors related to the immune system (i.e. leukocytes, B cells and T cells) in both the blood and spleen are observed with whole-body proton irradiation that can impact cancer immunotherapy [4]. In addition to clinical implications, increased knowledge of the impact of proton irradiation on host tissues will inform risk assessments for long-term space travel, since protons account for >80% of galactic cosmic rays (GCRs) and a large proportion of solar particle events (SPEs) [5, 6].

Epidemiological data show a near-exponential increase in cancer incidence starting from adolescence until middle age; thereafter, incidence starts to decelerate and in old age (>70 years) begins to decrease [7]. Recent publications demonstrate that age plays a major role in the modulation of tumor progression, with a reduced capacity of older hosts to maintain and promote tumor progression with further impact from proton irradiation [8–10]. Here we expand on these findings by examining the role of distant non-tumor-bearing host organs (e.g. spleen) in the integrated tumor–host system. For the following reasons, the spleen was examined in the tumor–host system as a function of proton irradiation and aging: (i) as the largest secondary lymphoid organ tasked with initiating immune responses [11], the spleen has been shown to be involved in tumor immune–escape mechanisms [12]; (ii) the spleen is highly radiosensitive [1] (proton radiation induces general splenic organ depression [4]); (iii) age impacts overall splenic morphology and function by changes occurring within the germinal centers and decreases in lymphocyte counts (11); and (iv) the spleen has been implicated as being modulated by the effects of radiation in distant organs and tissues [12, 13]. In summary, the spleen is an organ that should experience a large degree of functional perturbation in the host due to its mentioned sensitivity to the presence of a tumor–host system, proton radiation, and age.

The effects of whole-body irradiation with fractionated 1-GeV protons on splenic tissue from adolescent and old mice implanted with syngeneic Lewis lung carcinoma (LLC) cells were investigated. Differential gene expression patterns were observed that were strongly associated with modulation of both age and proton irradiation status. Several key factors were discovered which appeared to be specifically related to the global changes in host biologic phenotype and modulation of overall immune function and cell cycling, including MCM7, CDK2, RUVBL2 and CD74. An unbiased systems biology approach revealed global signaling changes detected in host spleens that impact the host capacity to support tumor progression.

## MATERIALS AND METHODS

### Cell culture

Murine Lewis lung carcinoma (LLC) cells, originally derived from a spontaneous tumor in a C57BL/6 mouse [14], were obtained from the American Type Culture Collection (Manassas, VA). The LLC cells were cultured under standard conditions [14] in high glucose DMEM (Gibco Invitrogen Cell Culture, Carlsbad, CA) with 10% FBS (Gibco Invitrogen Cell Culture) and 5% CO_2_.

### Tumor Injections

Tumor injections for this study were performed as previously described by Beheshti *et al.* (10). Injections of C57BL/6 mice were made subcutaneously in the mid-dorsal region of the back with 10^6^ LLC cells, 8 h after the first of three dose fractions. Unirradiated mice, serving as controls, were similarly injected with 10^6^ LLC cells. Mice were anesthetized by inhalation of 4% isoflurane, for injection of 10^6^ LLC cells suspended in 0.2 ml phosphate-buffered saline (PBS). Sham mice were treated identically. Scaling of mouse age to human age was accomplished using published criteria [15]. Age comparisons are as follows: mice at 68 days (adolescent mice) and 736 days (old mice) are considered approximately equivalent to 17- and 75-year-old humans. LLC tumors were only present in the injection site at the mid-dorsal region and were measured regularly (See Supplemental Fig. 1). No metastases or tumor growth appeared in other locations or organs. Subcutaneous LLC tumors take, on average, two weeks to reach 1.5 cm^3^, at which point the mice were sacrificed and the tissue processed.

### Irradiation of mice

Thirty C57BL/6 male mice 68 days old (Jackson Laboratory) and thirty 736 days old (National Institute of Aging) were used in this study, and irradiation protocols of these mice were previously published [10]. Ten adolescent and ten old mice were given whole-body irradiation once a day for three days with 0.5 Gy of proton ions (1-GeV; LET 0.24-keV/µm; plateau, non-Bragg peak, region, 0.5 Gy/min dose rate) at Brookhaven National Laboratory (BNL) (Upton, NY) (Supplemental Fig. 1). Mice were restrained and were not anesthetized during irradiation. An additional 20 adolescent and 20 old mice were not irradiated, but otherwise handled identically and used as sham, unirradiated controls. All animals, control and irradiated, were injected with syngeneic LLC cells after the first of three fractions of sham or proton irradiation. The timing of the dosing relative to cancer cell injection was chosen in order to maximize the likelihood of the impact of proton irradiation on tumor development and advancement. This was achieved by combining the situation where the first irradiation fraction acts on the normal cells of the host and later fractions modulate interactions between the host and the cancer cells.

### Tissue processing

Mice were monitored regularly, and tumor size was measured with calipers by a single individual. Once tumors reached ∼1.5 cm^3^, mice were sacrificed with a 0.6-ml intraperitoneal injection of 2,2,2-tribromoethanol at 20 mg/ml. Tissues to be frozen-sectioned were dissected and slow-frozen in OCT (Tissue Tek, Fisher Scientific, Pittsburgh, PA) in the gas phase of liquid nitrogen, and tissues for RNA isolation were snap-frozen in liquid nitrogen. Tissues to be paraffin-sectioned were placed in 10% formalin, processed by standard protocol [16], placed in cassettes, and paraffin embedded. Paraffin-embedded tissues were cut into 4-μm slices, placed on positively charged slides (Fisher Scientific), and stained with hematoxylin and eosin (H&E) stain using standard protocols [16].

### Antibodies

Details on the antibodies are available in the Supplemental Methods.

### Immunofluorescence staining on tissues

Standard immunofluorescent staining protocols were used as in previous publications [9]. More details appear in the Supplementary Methods.

### Westerns

Total protein was extracted and isolated from snap-frozen tissue by a standard extraction protocol, as previously published [9]. Standard western protocols were used and further details are available in the Supplemental Methods.

### Real-time quantitative PCR

RNA was isolated from splenic tissue in TRIzol (Invitrogen, Carlsbad, CA) using standard methods and homogenized using a Tissue Lyser II (Qiagen, Valencia, CA). Tissue with TRIzol was extracted according to the manufacturer's instructions, as was previously reported [8]. Probes for CD74, CDK2, RUVBL2, MCM7 and TGFβ1 were commercially available (Applied Biosystems, Carlsbad, CA). Assays were performed with technical duplicates and data were analyzed using the method described by Schmittgen and Livak [17].

### Transcriptome analysis

For genome-wide expression profiling of splenic tissue, Mouse WG-6 bead array chips (Illumina, San Diego, CA) were used. Methods for obtaining gene expression array data were previously reported [8–10]. For spleen replicates, 10 spleen samples for each condition (0 Gy adolescent, 0.5 Gy x 3 proton adolescent, 0 Gy old, and 0.5 Gy x 3 proton old), i.e. a total of 40 spleen samples, were used. The data was corrected through COMBAT batch correction [18], then quantile normalization was applied. Data was imported into MultiExperiment Viewer, MeV [19] for analysis. The statistically significant genes were determined by applying a one-way ANOVA with an adjusted Bonferroni correction with a false discovery rate (FDR) < 0.001 that resulted in a list of 2374 significant genes. Further details on the analysis are available in the Supplemental Methods section. The data discussed in this publication have been deposited in NCBI's Gene Expression Omnibus [20] and are accessible through GEO Series accession number GSE67243 (http://www.ncbi.nlm.nih.gov/geo/query/acc.cgi?acc=GSE67243).

### Statistical analysis

Student's *t*-tests were used for statistical analysis as appropriate. All *P*-values were calculated using two-tailed tests. Differences were considered statistically significant if *P* < 0.05. Error bars in the graphs represent standard error. Tumor growth quantification and growth rate calculations were previously described in a parallel study focused on the tumor dynamics [10].

## RESULTS

### Modulation of tumor dynamics in mice as a function of age and proton irradiation

**Fig. 1.**
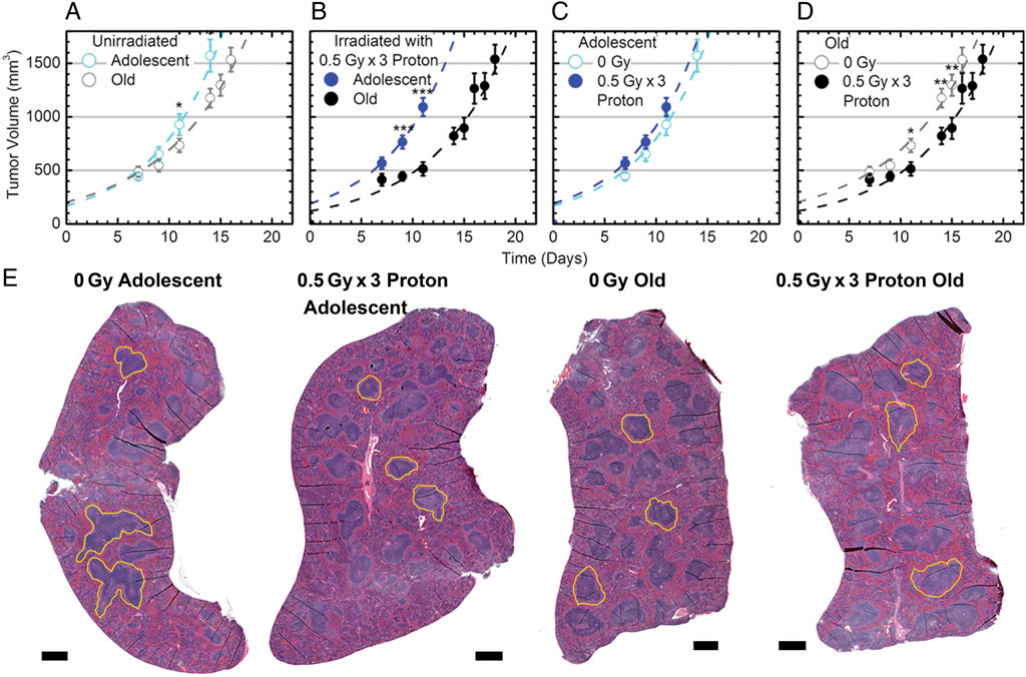
Tumor dynamics and morphological changes in spleens from tumor-bearing mice affected by age and 3 × 0.5 Gy proton irradiation. (**A–D)** The data show average measured tumor volumes, after injection at Time 0, of murine Lewis lung carcinoma (LLC) cells into C57BL/6 mice with and without 3 × 0.5 Gy of proton irradiation (1 GeV). This data is adapted from Beheshti *et al.* (10). The time post-cell-injection is shown on the *x*-axis. Error bars show ±SE; and asterisks indicate time-points where there is a statistically significant differential, even when ignoring information from the other time points, *P < 0.05, **P < 0.01 and ***P < 0.001. The dashed lines indicate generated best fits in terms of two quantities: tumor growth rates after approximately exponential growth starts, and ‘effective’ initial tumor volumes as explained in the Methods section. (**E**) Representative hematoxylin and eosin (H&E) stains on spleen samples. Black scale bar represents 500 μm. The yellow outline represents examples of the marginal zone in the spleen for each different condition.

The tumor growth dynamics displayed here were more extensively explored and investigated in Beheshti *et al.* [10]. All LLC tumor growth was observed only at the injection site (i.e. subcutaneous tumors), and no metastases were detected in other organs. The tumor dynamics showed no significant difference in tumor growth between the irradiated adolescent mice (AP) and the unirradiated adolescent mice (A) (Fig. 1C). All subsequent comparisons showed that tumor growth was significantly decreased in old irradiated mice (OP) versus old unirradiated mice (O) (Fig. 1D), in old unirradiated mice (O) when compared with adolescent unirradiated mice (A) (Fig. 1A), and in old irradiated mice (OP) when compared with adolescent irradiated mice (AP) (Fig. 1B). Hence, tumor growth was significantly reduced in comparisons where mice were either older (O vs A and OP vs AP) (Fig. 1A and B) or received irradiation (OP vs O) (Fig. 1D), with the exception of adolescent irradiated mice (AP vs A) (Fig. 1C).

### Morphological differences in the spleens of tumor-bearing mice as a function of age and proton irradiation

Representative H&E slides of each experimental group show physical differences between age groups and irradiation conditions. The marginal zones (the border regions of the white pulp which screen the systemic circulation for antigens and pathogens) [11] apparent in the adolescent unirradiated animals tended to be larger and more irregular than both the irradiated adolescents and the old unirradiated animals (represented by the yellow outlines in Fig. 1E). Qualitatively, the old unirradiated and old irradiated spleens appear very similar (Fig. 1E). Importantly, there was no neoplastic activity evident in any of the cross-sections displayed, and thus all downstream analysis is likely to reflect only activity of normal host tissue without metastatic lesions.

### Global transcriptome changes in spleens of tumor-bearing mice caused from both aging and proton irradiation

**Fig. 2.**
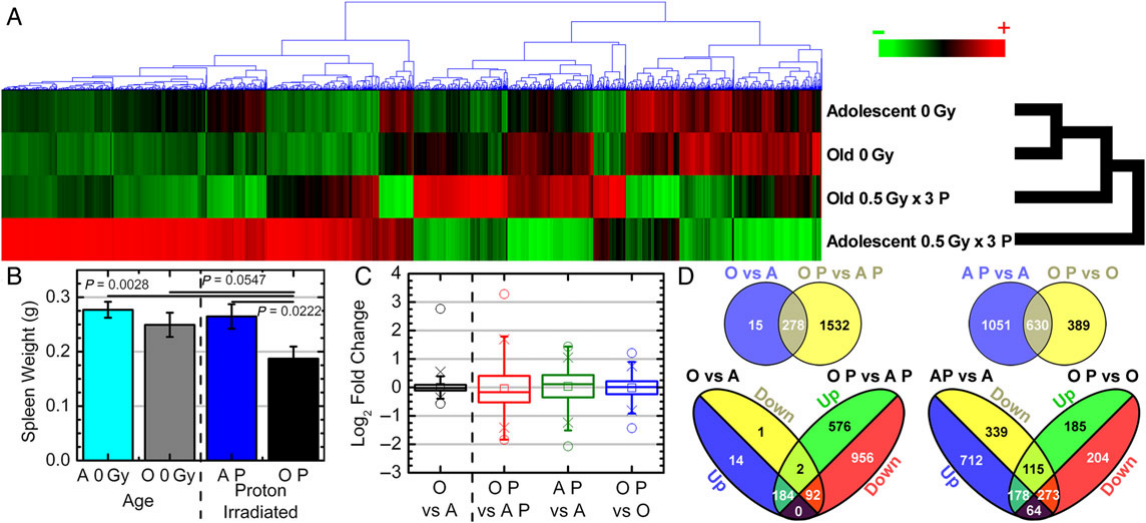
Gene regulation and physical changes for spleens as a function of host age. (**A**) Hierarchical clustering of genes by average linkage (UPGMA) and Euclidean distance calculation in the spleen with different age and proton irradiation for the 2374 significant genes with one-way ANOVA, FDR < 0.001. (**B**) Weights of spleens from C57BL/6 male mice with different age and proton irradiation comparisons (0 Gy adolescent (A) *n* = 20, 0 Gy old (O) *n* = 20, 0.5 Gy x 3 proton adolescent (AP) *n* = 10, 0.5 Gy x 3 proton old (OP) *n* = 10). (**C**) Average signal log_2_ fold-change comparing 0 Gy adolescent (A), 0 Gy old (O), 0.5 Gy x 3 proton adolescent (AP), 0.5 Gy x 3 proton old (OP) with each other. Whiskers show the range of the outliers, with max and min values as 0 and the 1 and 99th percentile outliers as X. (**D**) Venn diagrams of the genes with 1.2-fold change for comparisons between adolescent and old spleen samples with and without proton irradiation, with separate Venn diagrams for only the up- and downregulated genes.

Transcriptome analysis of the spleens from the mice described above demonstrates clear differences between the various age and radiation groups. Significantly regulated genes with a FDR < 0.001 were determined, resulting in 2374 genes. Euclidean cluster analysis of the significantly regulated genes clearly shows that overall gene expression data in proton-irradiated adolescent hosts is significantly different from all other groups, while the old and adolescent non-irradiated groups were the most similar (Fig. 2A). Transcriptome changes in the spleen mimic morphological differences, with the greatest difference appearing between the proton-irradiated adolescent and unirradiated adolescent spleens (Figs 1E, 2A and 2C). The irradiated adolescent mice (AP) vs unirradiated adolescent mice (A) and irradiated old mice (OP) vs AP comparisons had the largest variability respectively, while the OP vs old mice (O) and O vs A had the least variability (Fig. 2C). This was not reflected by spleen physical characteristics in terms of mass, as only the old irradiated group was significantly smaller than the other three groups (Fig. 2B). An overall upregulation in significant genes existed for the AP vs A group (954 upregulated vs 727 downregulated), while radiation had only a minimal overall downregulation in the OP vs O group (541 downregulated vs 478 upregulated) (Fig. 2D). Interestingly, 179 genes were oppositely regulated by radiation for both AP vs A and OP vs O (64 upregulated in AP vs A and downregulated in OP vs O and 115 downregulated in AP vs A and upregulated in OP vs O). As a function of age, only two genes between the O vs A and OP vs AP were oppositely regulated. O vs A shows a very modest overall upregulation of key genes (198 upregulated vs 95 downregulated), while OP vs AP shows an overall downregulation (1048 downregulated vs 760 upregulated) (Fig. 2D).

### Global biological changes as a function of age and proton irradiation in the spleen

**Fig. 3.**
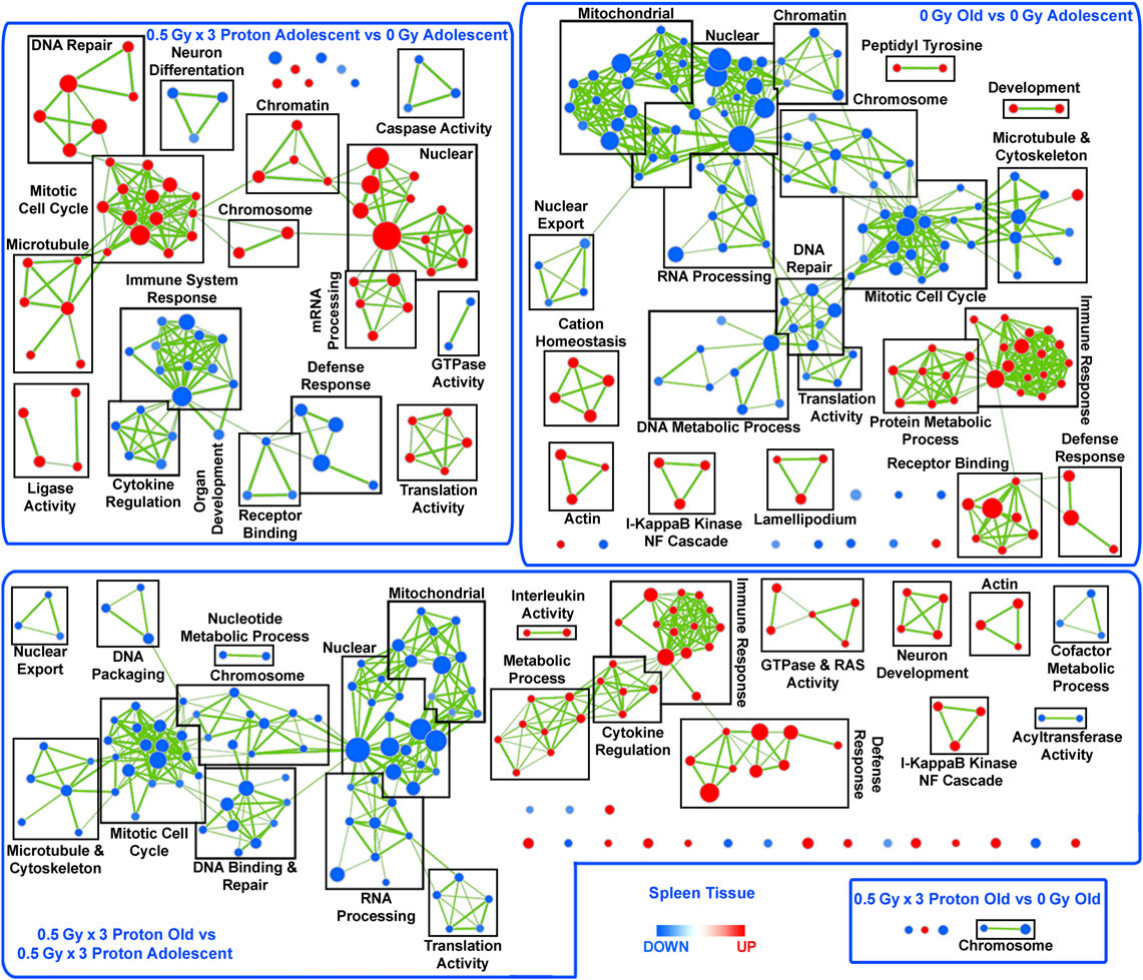
Network representation of Gene Set Enrichment Analysis (GSEA) for GO C5 gene sets. Leading edge analysis with a FDR < 0.05 determined significant gene sets enriched for each group. The size of each node reflects the number of molecules involved for each gene set. The edge (green lines) represents the number of genes associated with the overlap of two gene sets (or nodes) that the edge connects. Clusters were named according to common function in each grouping. Upregulated gene sets were denoted with red color, and downregulated gene sets were denoted by blue color.

A list of Gene Ontology (GO) categorized functions were determined through GSEA (Supplemental Table 1) and was visualized as a network (Fig. 3), where clusters were identified according to common significant gene sets (FDR < 0.05). DNA repair, mitotic cell cycle, and nuclear and mRNA processing were significantly upregulated in the irradiated adolescent compared with the 0-Gy adolescents, while general immune system response, defense function and cytokine regulation were downregulated (Fig. 3). Additionally, the comparison of old with adolescent irradiated mouse spleens had the opposite effect, where functions related to cell cycle, mRNA processing, and DNA repair were downregulated, while functions related to inflammation and the immune response were significantly upregulated (Fig. 3). A very similar pattern also appeared in the non-irradiated spleens from old versus adolescent mice (Fig. 3). It should also be noted that the degree of up- or downregulation within each node are not identical for OP vs AP and O vs A groups, indicating that the addition of radiation to the system (OP vs AP) created a unique host response in comparison with the non-irradiated system (O vs A) (Supplemental Table 1). Minimal differences were observed for the spleens comparing old mice with or without proton irradiation (Fig. 3). Overall as a result of proton irradiation, immunosuppression, increased cell cycling, and DNA repair occurs primarily in adolescent hosts; while opposite effects occur for old hosts without proton irradiation.

Further analysis was performed using Ingenuity Pathway Analysis (IPA) software to determine key molecular differences occurring between each group. Biofunction analysis through IPA confirms the GSEA results, revealing decreases in immune functions for AP vs A related to cell movement, chemotaxis, quantity and homing, while functions related to mitogenic responses such as cell cycling and replication are increased (Supplemental Table 2). These biofunctions are also oppositely regulated in the old vs adolescent irradiated spleens and bolster the patterns observed from the GSEA analysis.

**Fig. 4.**
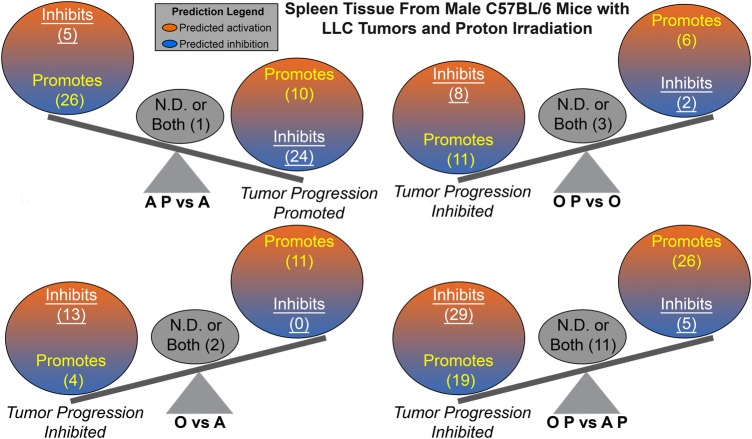
The impact of predicted Upstream Regulators from the spleen on tumor progression. A schematic of the activation states of the upstream regulators from Supplemental Table 3 illustrating the balance between the tumor promoters (text in yellow) and tumor suppressors (text in white and underlined), with a predicted activation (orange) or predicted inhibition (blue). Upstream regulators with both promoter and suppressor effects or the effects have not been not determined (N.D.) are shown in the middle of the balance (gray circle and black text). The number of upstream regulators per group is indicated in the parentheses.

In order to further understand the tumor host environment, the differences in expression of multiple upstream regulators were investigated. Upstream regulators were determined using IPA and were defined as any molecule that affected the expression or function of another molecule (Supplemental Table 3). Once again the AP vs A when compared with OP vs AP or O vs A was oppositely regulated (Supplemental Table 2). Tumor progression was predicted from the upstream regulators by determining from the literature whether each upstream regulator promotes or inhibits tumor progression. It was predicted that a tumor-promoting environment occurs in the spleens from adolescent irradiated tumor-bearing mice, while tumor inhibition is predicted in all other comparisons (Fig. 4). Although the balance between the tumor promoters and the suppressors are rather close in numbers for AP vs A, the overall prediction for tumor promotion is consistent with the biology presented throughout this manuscript.

### Immune cell subset modulations within the spleen

**Fig. 5.**
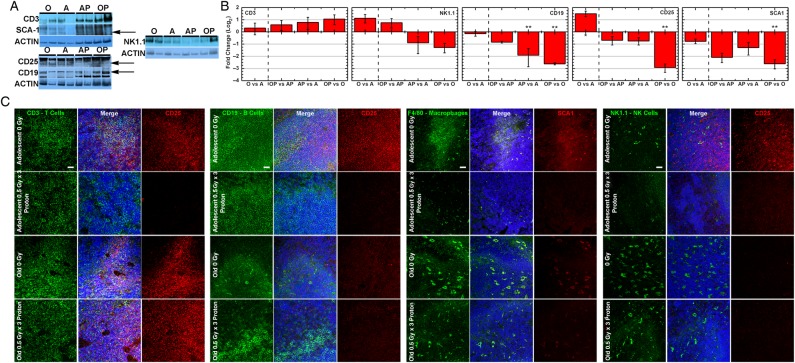
Immune cell subset identification as a function of age for proton-irradiated hosts versus unirradiated hosts on spleen tissue. (**A**) Representative western blots for specific markers for different immune subsets and the associated activation marker. Arrows indicate the location of the correct band. (**B**) Quantification of the western blots for each marker and condition. For each condition a total of *n* = 4 was used. Significant differences are indicated by *P < 0.05, **P < 0.01. (**C**) Representative immunofluorescent staining for each condition for antibodies associated with specific immune cell subsets and the associated activation marker. White scale bar = 20 µm.

To identify which components of the spleen are involved with the observed immune function changes found in the GSEA networks, a phenotypic evaluation of immune cell subsets was determined. Immune subset activation was characterized through identification of CD25 which is an activation marker for B, T, and NK cells [21, 22] and SCA1 which represents activated macrophages [23]. On examining all AP vs A comparisons, there was a clear decrease in immune cell activation in all subsets when CD25 is co-localized with T, B, or NK cells (Fig. 5C), while there was minimal if any change in activation of these cells in OP vs O for T cells (CD3 co-localization) (Fig. 5C). Interestingly the overall activity for B, T, and NK cells is increased for old unirradiated hosts compared to adolescent hosts (which is in agreement with the GSEA results (Fig. 3)), indicating that the overall immune activity of tumor bearing old mice is greater than adolescent hosts (Fig. 5B). Additionally, fewer NK cells (identified through NK1.1 [24]), macrophages (identified through F4/80 [25]), and B cells (identified through CD19) exist after proton irradiation in both adolescent and old hosts, indicating that regardless of age, proton irradiation causes a decrease in NK cells, macrophages, and B cells (Fig. 5). NK cells also increased with old age (O vs A and OP vs AP) independent of proton irradiation (Fig. 5), suggesting that old tumor bearing hosts have increased immune function. Macrophages could not be detected by western blot, due to the low amount present in the spleen for all conditions as indicated by immunofluorescence (Fig. 5C). A similar pattern occurs for activated macrophages (SCA1) with a decrease in activity for proton irradiation independent of age (Fig. 5). It should be noted that while the Ly-6 protein family members [23] and specifically SCA1 (Ly-6A/E) [26] has been linked to macrophage activation, it has an additional major role as an antigenic marker of stem and progenitor cells, and is thought to decrease as these cells differentiate [27]. Thus while SCA1 indicates macrophage activation when co-localized with the macrophage antigenic marker F4/80, independent quantitation of SCA1 by western blot does not reflect macrophage activation levels alone.

Old hosts independent of irradiation appear to have a greater amount of NK cells and have significantly higher T, B, and NK immune cell activity (Fig. 5B). Additionally proton irradiation increases the amount of T cells in spleens from old hosts (Fig. 5). This plays an important role in the overall activity when comparing to adolescent irradiated spleens. Overall T cell activation may be decreased in OP vs O (Fig. 5B), but direct T cell associated activation (CD25 positive cells co-localized with CD3 positive cells) in AP is almost entirely absent when compared to A while no change occurs for OP vs O (Fig. 5C). Specific analysis from the immune cell subsets combined with immune system changes determined from transcriptome analysis, demonstrates immunosuppression occurring in spleens from proton irradiated tumor-bearing adolescent hosts due to T cell activity. Overall the immunosuppression for adolescent irradiated hosts can be attributed to reduction of T cell immune activity in combination with the decreases in other immune subset numbers and activity.

### Key genes involved in the differences in spleens as a function of age and proton irradiation

**Fig. 6.**
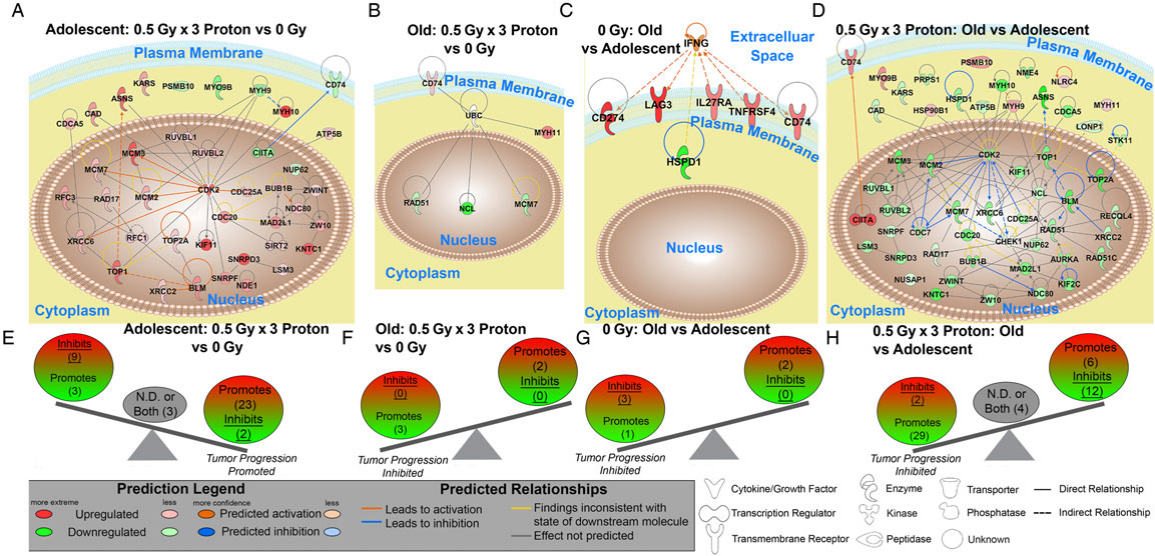
Gene network analysis for the key genes involved in splenic changes. (**A–D**) Pathway analysis was done with Ingenuity Pathway Analysis (IPA) software. IFNG and UBC were added to specific networks to provide relations and connections between all genes. Predicted relationships between all genes are also shown. Log_2_ fold changes to the gene expression were used to obtain different shades of green for regulation levels for 1.2-fold change in downregulated genes, while different shades of red depict regulation levels for 1.2-fold change in upregulated genes. The darker the shade of green or red, the greater the fold change. (**E–H**) A schematic of the key significant genes (Supplemental Table 4), determined to be significant in regulating many functions for each comparison, illustrating the balance between the tumor promoters and tumor suppressors with the Log_2_ fold change color coded as before. Genes with both promoter and suppressor effects, or the effects not determined (N.D.), are shown in the middle of the balance (gray circle and black text). The number of genes per group is indicated in parentheses.

A novel unbiased systems biology analysis previously established [9] was implemented to determine the key genes involved in the overall dynamics. The key genes were determined by finding the commonalities between the significant upstream regulators, the biofunction analysis, GSEA for GO gene sets, and DAVID functional annotation clusters (Fig. 6A–D and Supplemental Table 4). DAVID Gene Function Classification Tool measures the relationships among different biologically annotated terms based on the degrees of co-association of genes [28] and only genes in the top 10 functional annotation clusters were considered (Supplemental Tables 5–8). Tumor predictions from the key genes (Fig. 6E–H) were analyzed similarly as the upstream regulator tumor predictions (Fig. 4), with predicted tumor inhibition occurring in all groups except for the adolescent comparison, where tumor progression is promoted (Fig. 6E).

From the key genes, MCM7 and CD74 were in common between the majority of the comparisons and CDK2 and RUVBL2 were shown to regulate the majority of the other key genes when present (Fig. 6A–D). CD74 is required for B-cell differentiation, influences T-cell selection in the thymus, and functions as part of the macrophage migratory inhibitory factor (MIF) complex through which it participates in the inflammatory response [29, 30]. MCM7 is a part of the MCM complex involving MCM2-7 which acts as an essential part of DNA replication initiation and elongation, and is involved in S phase checkpoint signaling in response to DNA replication stress and damage via interaction with Rad17 at the site of a stalled replication fork [31]. CDK2 is part of a family of serine/threonine kinases [32] that act in concert with a corresponding cyclin (in this case cyclin E or cyclin A) to control the progression of the cell cycle, DNA synthesis, and DNA replication [33]. CDK2 activation by a heterodimerization with cyclin E (the Cdk2-CycE complex) phosphorylates Rb, releases E2F, which in turn initiates DNA synthesis [33, 34]. Finally, RUVBL2 is an ATPase associated with diverse cellular activities involved in chromatin remodeling, DNA repair, regulation of transcription, ribonucleoprotein assembly [35], cell growth, and cell migration and invasion [36].

**Fig. 7.**
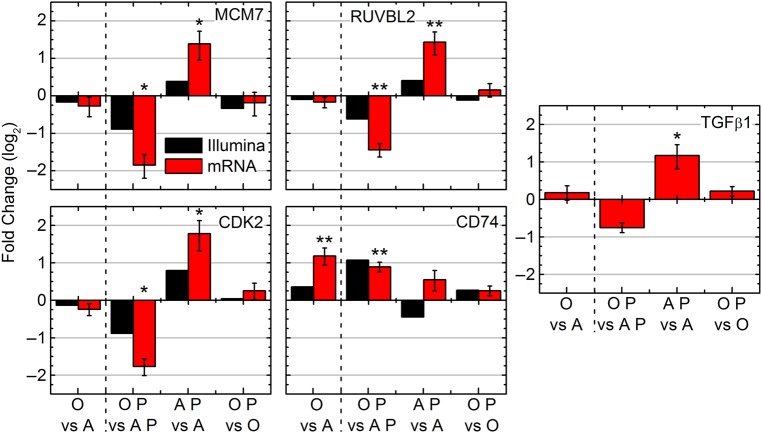
Significant molecular factors involved in the spleen with different age and proton irradiation comparisons (0 Gy adolescent (**A**), 0 Gy old (O), 0.5 Gy x 3 proton adolescent (A P), 0.5 Gy x 3 proton old (O P)). The mRNA expression determined by real-Time PCR (RTPCR) for MCM7, RUVBL2, CDK2, CD74 and TGFβ1 compared with the gene expression observed by Illumina. Log_2_ fold changes for mRNA were determined for each group, *P < 0.05 and **P < 0.01.

The array data demonstrates that MCM7, CDK2, and RUVBL2 were upregulated for AP vs A and downregulated in the rest (with the exception of CDK2 in the OP vs O category) while CD74 was shown to be downregulated for AP vs A and upregulated in the other three. To validate these findings, quantitative real-time PCR (RT-PCR) was performed on these four genes (Fig. 7). In agreement with the array data, MCM7, CDK2, and RUVBL2 were all significantly increased for AP vs A, while being significantly downregulated for OP vs AP. RT-PCR, however, reveals that CD74 is upregulated in all groups, while the expression data shows CD74 being downregulated for AP vs A, indicating that a false positive occurred. As mentioned previously, the FDR for the gene expression analysis was less than 0.001, meaning that one out of every thousand genes would have potential to be falsely regulated. Both low signal intensities and background noise contribute to the detection of false positives in microarray data [37], and one or both issues could have occurred to produce the false positive in the present case. CD74 however, is clearly significantly upregulated in both age comparison groups, regardless of irradiation status.

Finally, expression levels of TGFβ1 in the spleens were determined. Previously published results demonstrated from transcriptome analysis on the tumor that TGFβ1 was a key molecule involved in the tumor dynamics [10]. Suppression of TGFβ was associated with tumor growth inhibition in proton-irradiated old mice [10]. A similar pattern of splenic mRNA TGFβ1 levels (Fig. 7) was shown compared to MCM7, CDK2, and RUVBL2, with a significant increase for AP vs A and downregulation for OP vs AP.

## DISCUSSION

Age and proton irradiation modulations of the overall tumor-host dynamic have important implications for both proton radiotherapy and galactic space radiation exposure. This analysis studied how the host spleen tissue of tumor-bearing mice reacted to proton whole body irradiation at 3 × 0.5Gy (1 GeV, LET = 0.24 keV/μm; plateau, non-Bragg peak, region) on adolescent and old mice after the establishment of a tumor.

It is well known that damage to radiosensitive organs, like the spleen or bone marrow, leads to immunosuppression [1, 38]. Heterogeneous effects of proton irradiation on splenocyte immune cell populations occur [1] and while there is an overall immune depression, a larger effect on splenic lymphocytes than on splenic monocytes/macrophages and granulocytes is observed [39]. Overall immune system function was affected in this study; however the changes were surprisingly not uniform over all comparisons (Figs 3 and 5). Importantly, it appears that proton irradiation downregulated immune function in adolescents, in agreement with the current knowledge about splenic radiosensitivity. Surprisingly, this modulation was absent for T cells in irradiated old subjects (Figs 3 and 5). This was corroborated by the decrease in immune cell activation in B, T, and NK cells and fewer NK cells (Fig. 5B) macrophages (Fig. 5C), and B cells (Fig. 5B) in AP vs A with limited perturbation in OP vs O for T cell activity (Fig. 5C). When looking at modulation of age, independent of radiation (Fig. 3), it appears that old age caused a general state of increased immune response due to T cell presence and activity (Figs 3 and 5). Thus, old hosts appear generally more immunologically resistant as a function of age.

Another important pattern was the dysregulation found in overall cell cycling and DNA repair (Fig. 3). These functions are downregulated independent of radiation for OP vs AP and O vs A, which is in agreement with the reported overall state of increased cellular senescence due to telomere shortening or increased volume of chronic DNA damage as a function of increasing age [40]. It also agrees with the evidence that DNA repair becomes less efficient due to stressful insults with age [41]. As mentioned above, the OP vs O comparison showed very little genetic dysregulation as a result of radiation, however the AP vs A comparison showed a general upregulation of overall cell cycling and DNA repair. These findings are in agreement with previous work, indicating that an acute dose of protons at 1.0 Gy or 2.0 Gy causes upregulation in multiple DNA repair genes with a proportional degree of increase in expression as a function of increasing dose [42]. Increased DNA synthesis has also been noted in splenic leukocytes following proton irradiation, where Gridley *et al.* hypothesize that this was due to activation of hematopoiesis to compensate for cell loss [43]. It seems likely that the same process is taking place in the adolescent irradiated hosts in this study as well. Although the spleens from old irradiated hosts did not show this, it is postulated that activation of hematopoiesis may have been competing with downregulation of cell cycling and DNA repair as a result of age (as stated above) and manifested in the minimal perturbation observed (Fig. 3).

An overall pattern of inhibited tumor progression was predicted in the spleens from tumor-bearing hosts in all comparisons except for proton irradiated adolescent hosts compared to unirradiated adolescent hosts, which predicts tumor promotion (Figs 4 and 6). These predictions remarkably are in agreement with the previous study only focusing on the tumor from the same mice (10). Tumor progression was inhibited in O vs A, OP vs AP, and even in OP vs O (Fig. 1A, B and D), with each of these comparisons reaching statistical significance. Altogether this would indicate that the tumor and host must be influencing each other on a genetic level. From the spleen transcriptome data increased tumor growth for AP vs A was predicted (Figs 4A and 6E), but in fact there was no difference in growth observed (Fig. 1C). It is hypothesized that the anti-cancer properties specific to proton radiation (anti-angiogenesis, diminished tumor growth, and impaired cell invasion [2, 3]) directly effecting the tumor, must have been counteracting the tumor promoting environment discovered here to generate the observed growth properties.

CD74 was upregulated by age (regardless of the addition of radiation) (Fig. 7) in agreement with the literature [44]. Upregulation of CD74 is also associated with upregulation of inflammation and general immune pathways [29, 30], and this also agrees with the analysis for O vs A and OP vs AP (Fig. 3). More work is needed to elaborate CD74's key role in ‘inflammaging’ and its potential role as a buffer to radiation induced immunosuppression noted in older subjects.

The genes *CDK2*, *MCM7* and *RUVBL2* on the other hand, are all involved in DNA repair and cell cycling as mentioned above, and their expression levels were observed to follow the same patterns (upregulated in AP vs A and downregulated in OP vs AP). CDK2 plays a major role in controlling progression of the cell cycle [33], and interestingly has been found to be sensitive to exposure to reactive oxygen species (ROS). In the spleen, ROS has been shown to act at mitogenic stimulators, and result in hyperproliferation through increased CDK2 expression [34]. This mechanism appears to occur in irradiated adolescent hosts when compared to unirradiated adolescent hosts indicated with increased CDK2 expression and increased cell cycling in response to proton radiation (Figs 3 and 7). Downregulation for OP vs AP was observed as a result of decreased cell cycling (Fig. 3). In combination with the observation of a lack of CDK2 dysregulation for OP vs O, this pattern suggests an overall environment for increased tumor progression for proton irradiated adolescent hosts due to overall increased cell cycling. MCM7 has also been shown to have a strong ability to promote cell cycle progression and with strong involvement in human cancers [45] with overexpression inducing improper DNA synthesis [46]. Finally, numerous studies have shown a critical role of RUVBL2 in controlling cell growth [36] with overexpression responsible for oncogenesis in mixed lineage leukemia and promotion of metastatic disease [35]. Additionally, RUVBL2 responds to ionizing radiation through interaction with the transcriptional regulator ATF2 to increase DNA repair of double stranded-breaks, and to prevent both cell cycle arrest and apoptosis [47], identical to the pattern displayed when comparing proton irradiated adolescent hosts to unirradiated adolescent hosts (Fig. 3). The key genes, CDK2, MCM7, and RUVBL2, have all been observed to have an increased level of expression for adolescent hosts irradiated with protons (Figs 6A and 7), which likely contribute to the picture of an overall environment predisposed to both oncogenesis and tumor promotion (Figs 4A and 6E). All three genes were minimally perturbed by proton radiation in old hosts compared to unirradiated hosts and this is likely responsible for the genotype dysregulation found when comparing OP vs AP. The addition of radiation to aged hosts causes a larger genetic disparity than the aging of the hosts alone. This is attributed to the overall reduced cell cycling found in old age, and the contrasting ability of adolescent subjects to respond to the radiative insult.

**Fig. 8.**
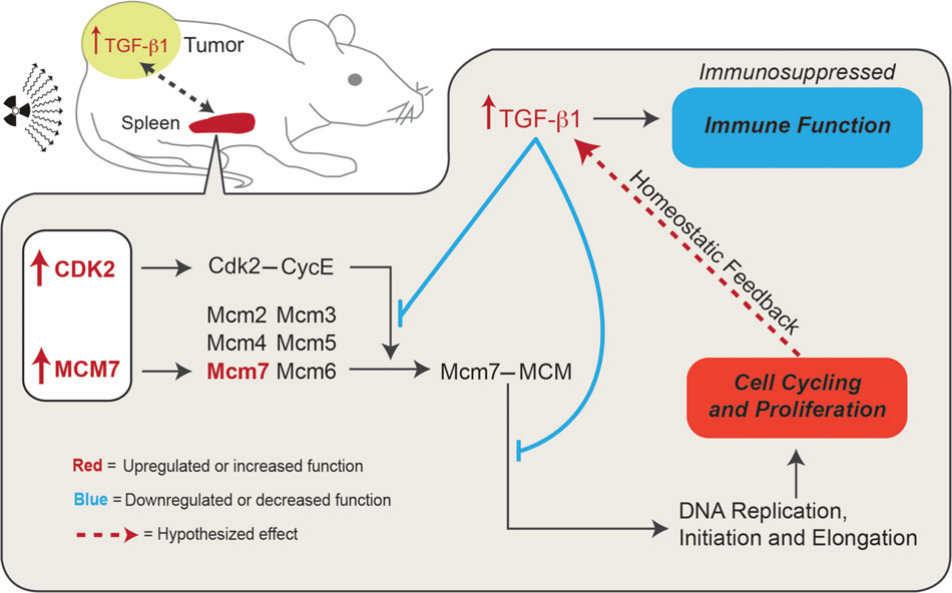
Hypothesized TGFβ1 homeostatic feedback mechanism occurring within the proton-irradiated adolescent (AP) hosts. All genes, functions or interactions that are upregulated are color-coded red, while all functions or interactions which are downregulated or inhibitory are color-coded blue. The hypothesized interactions in the spleen are denoted by a red dotted line. The gray dotted line represents the hypothesized interaction between the spleen and the tumor.

Lastly, expression levels of TGFβ1 in the spleens were determined because of their relevance to the previous study performed directly on the tumor [10]. The TGFβ1 expression pattern in the spleen of tumor-bearing mice closely mirrored that of the recognized key genes (Fig. 7). TGFβ1 is important for maintaining homeostatic control of growth in premalignant and early-stage carcinogenic cells, which allow TGFβ1 to act as a tumor suppressor [48]. TGFβ expression however, has paradoxically been shown to enhance tumor growth for late stage tumors [49]. Tumors used in this study are considered late-stage tumors due to the size at time of harvest. This is not necessarily the effect it would have on the splenic non-cancerous host tissue, and in fact TGFβ should be creating an inhibitory proliferative effect in the AP vs A comparison given its increased level of expression (Fig. 7). Mukherjee *et al.* observed that TGFβ1 has inhibitory effects on both CDK2 and MCM7, causing cell cycle downregulation in early G1 via inhibition of CycE-Cdk2, while TGFβ1 exposure in late G1 causes deactivation of the MCM complex via MCM7, also leading to cell cycle arrest (50). Thus, if TGFβ1 is elevated for proton irradiated adolescent hosts (Fig. 7), then cell cycling (and thus the actions of MCM7 and CDK2) should be diminished, however the genetic dysregulation is instead the opposite (Figs 3 and 7). The key here is that while Mukherjee et. al. first demonstrated the inhibitory effects of TGFβ1 on CDK2 and MCM7, they also showed that overexpression of MCM7 actually abrogates cell cycle arrest by TGFβ1 [50]. Thus, since the key genes of MCM7, CDK2, and RUVBL2 are: (i) indicated as the major players (and the starting point) in the dysregulation caused by the presence of a tumor and proton irradiation in an adolescent host; (ii) are those that ultimately led to a picture of increased cell cycling; and that (iii) TGFβ1 functions to maintain homeostatic control of cell cycling and proliferation; it seems more than likely that TGFβ1 is responding to an increased proliferative picture via a feedback loop inhibition which is abrogated by the high levels of the key genes identified (Fig. 8). It is important to note that TGFβ1 is also known as an immunosuppressive cytokine that has broad inhibitory effects on T-cell proliferation and activation, cytotoxic T lymphocyte cytotoxic program, antigen presentation by antigen presenting cells [51], and overall contributes to the immunological escape of tumor cells [52]. This would also be consistent with the near complete absence of T cell activation in AP vs A (Fig. 5C). Thus, the increased level of TGFβ1 expression found for AP vs A likely contributed to the overall picture of immunosuppression (Figs 3 and 5). Additionally, TGFβ1 contributed to the overall tumor-promoting environment (Figs 4 and 6E) through diminished anti-tumor immune activity, and could act as a side-effect via the postulated feedback loop (Fig. 8) created by the increased level of proliferation in the splenic tissue. Finally, since the tumor growth dynamics (Fig. 1A–D) are consistent with the predicted level of tumor progression or inhibition in the spleen (Figs 4 and 6), it can be hypothesized that the elevated levels of TGFβ1 found in the adolescent irradiated tumor tissue [10] and in the adolescent irradiated spleen (Fig. 7) is due to communication occurring between these tissues (Fig. 8).

In conclusion, age modulation was found to significantly alter transcriptome expression in a host-tumor system when perturbed by whole body proton irradiation. Older tumor-bearing subjects showed resistance to immunosuppression through T cell functions, which provided global splenic host changes impacting carcinogenesis, and overall genetic differences after proton irradiation. Proton irradiation in adolescent hosts appeared instead to set off a reaction resulting in host cell proliferation, DNA repair, and immunosuppression, all leading to overall splenic signaling predisposed to further carcinogenesis. Future research is needed to determine the threshold age which older subjects acquire resistance to tumor progression. Improved understanding of the risk groups could lead to better estimates for risk of carcinogenesis and help inform age-group based cancer screening for both the space radiation and radiation oncology communities. This study has also suggested a number of key factors that may be indicative of disease state, and requires further study for validation of this association. If this is accomplished, these factors could aid in identification of those with subclinical tumor progression after known proton radiation exposure.

## SUPPLEMENTARY DATA

Supplementary data is available at the *Journal of Radiation Research* online.

## FUNDING

Funding for this work was supported by the National Aeronautics and Space Administration under NSCOR Grant Nos NNJ06HA28G and NNX11AK26G issued through the Human Research Program to LH and by Award Number U54CA149233 from the National Cancer Institute, to LH. Funding to pay the Open Access publication charges for this article was provided by The Cancer Research Innovation Fund at Tufts Medical Center to AME.

## Supplementary Material

Supplementary Data
