# Supplementary material for: Proton irradiation impacts age-driven modulations of cancer progression influenced by immune system transcriptome modifications from splenic tissue
Source: J Radiat Res. 2015 Aug 7;56(5):792–803. doi: 10.1093/jrr/rrv043 (PMC4577010; doi:10.1093/jrr/rrv043)
Supplement: Supplementary Data [file supp_56_5_792__index.html]

Proton irradiation impacts age-driven modulations of cancer progression influenced by immune system transcriptome modifications from splenic tissue — Proton irradiation impacts age-driven modulations of cancer progression influenced by immune system transcriptome modifications from splenic tissue — Supplementary Data 

# Proton irradiation impacts age-driven modulations of cancer progression influenced by immune system transcriptome modifications from splenic tissue

## Supplementary Data

Supplementary Data

- Supplementary Figure 1 - docx file
- Supplementary Table 1 - doc file
- Supplementary Table 2 - doc file
- Supplementary Table 3 - doc file
- Supplementary Table 4 - doc file
- Supplementary Table 5 - doc file
- Supplementary Table 6 - doc file
- Supplementary Table 7 - doc file
- Supplementary Table 8 - doc file
