# Supplementary material for: Proton irradiation impacts age-driven modulations of cancer progression influenced by immune system transcriptome modifications from splenic tissue
Source: J Radiat Res. 2015 Aug 7;56(5):792–803. doi: 10.1093/jrr/rrv043 (PMC4577010; doi:10.1093/jrr/rrv043)

**Supplemental Figure Captions**

**Supplemental Figure 1.** Schematic of the mice that received whole-body proton irradiation, with the location of the tumor growth.


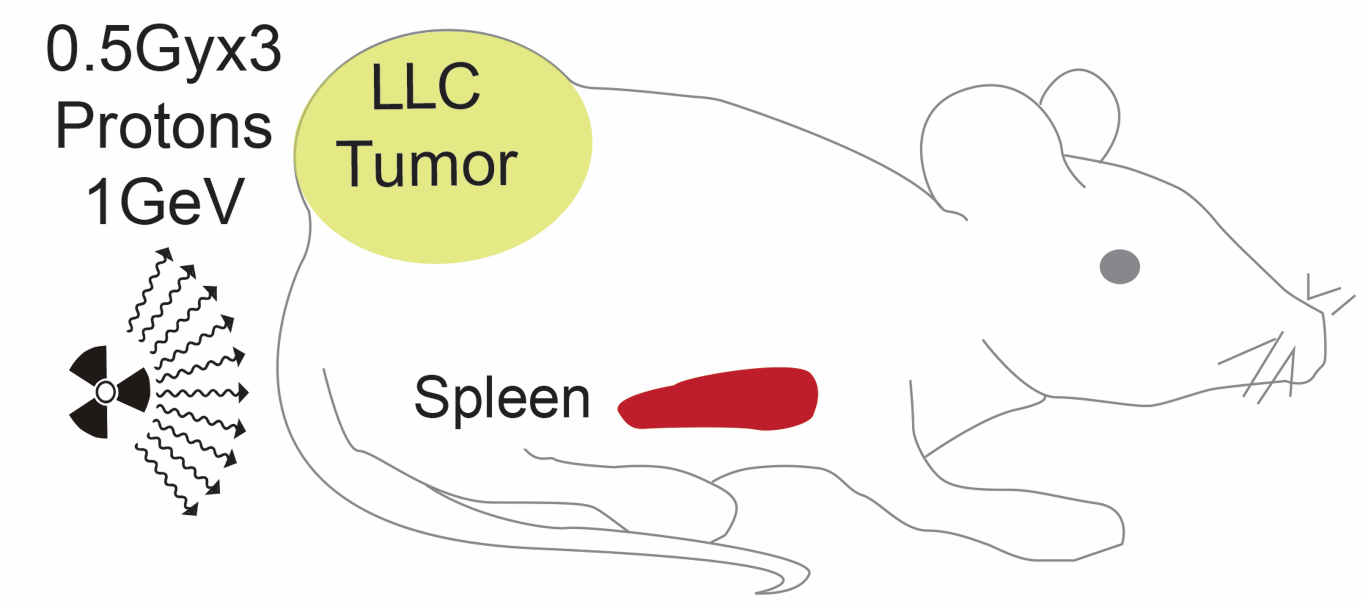

Supplement: Supplementary Data [file supp_rrv043_rrv043supp_fig1.docx]
