# Supplementary material for: Proton irradiation impacts age-driven modulations of cancer progression influenced by immune system transcriptome modifications from splenic tissue
Source: J Radiat Res. 2015 Aug 7;56(5):792–803. doi: 10.1093/jrr/rrv043 (PMC4577010; doi:10.1093/jrr/rrv043)
Supplement: Supplementary Data [file supp_rrv043_rrv043supp_table1.doc]

| **C5 Gene Set Enrichment** | **NES (# of Leading Edge Genes)** | | | |
| --- | --- | --- | --- | --- |
|  | **A P vs A** | **O P vs O** | **O vs A** | **O P vs A P** |
| Chromosome | 1.72 (38) | -1.93 (42) | -2.47 (43) | -2.02 (43) |
| Condensed Chromosome | 1.59 (14) | -1.96 (20) | -2.10 (15) | -1.95 (16) |
| Axonogenesis | -1.65 (5) | --- | 1.74 (7) | 1.70 (10) |
| Cell Activation | -1.97 (25) | --- | 2.15 (24) | 1.98 (28) |
| Cell Cycle Checkpoint GO 0000075 | 1.86 (18) | --- | -1.69 (16) | -1.82 (18) |
| Cell Cycle GO 0007049 | 2.00 (95) | --- | -2.34 (103) | -2.19 (125) |
| Cell Cycle Phase | 2.02 (54) | --- | -2.45 (54) | -2.24 (73) |
| Cell Cycle Process | 2.12 (69) | --- | -2.55 (64) | -2.31 (87) |
| Cellular Defense Response | -1.81 (13) | --- | 1.95 (11) | 1.86 (13) |
| Centrosome | 1.68 (19) | --- | -1.99 (21) | -1.75 (25) |
| Chemokine Activity | -1.75 (10) | --- | 2.18 (10) | 1.86 (11) |
| Chemokine Receptor Binding | -1.77 (10) | --- | 2.16 (10) | 1.87 (11) |
| Chromatin Assembly Or Disassembly | 1.70 (6) | --- | -1.92 (19) | -1.59 (18) |
| Chromosomal Part | 1.60 (27) | --- | -2.35 (35) | -1.89 (37) |
| Chromosome Organization And Biogenesis | 1.83 (14) | --- | -2.14 (13) | -1.96 (17) |
| Chromosome Segregation | 1.60 (36) | --- | -1.87 (47) | -1.91 (51) |
| Chromosomepericentric Region | 1.66 (15) | --- | -2.19 (16) | -1.84 (16) |
| Cytokine Biosynthetic Process | -1.80 (16) | --- | 1.93 (19) | 1.85 (13) |
| Cytokine Metabolic Process | -1.81 (16) | --- | 1.95 (19) | 1.85 (13) |
| Cytokine Production | -1.65 (22) | --- | 1.96 (30) | 1.81 (18) |
| Defense Response | -2.08 (63) | --- | 1.86 (51) | 1.94 (64) |
| DNA Dependent DNA Replication | 1.69 (19) | --- | -1.96 (19) | -1.93 (25) |
| DNA Metabolic Process | 1.88 (81) | --- | -2.10 (86) | -2.01 (101) |
| DNA Packaging | 1.75 (11) | --- | -1.85 (8) | -1.77 (13) |
| DNA Recombination | 1.62 (15) | --- | -1.9 (13) | -1.73 (18) |
| DNA Repair | 1.84 (50) | --- | -2.04 (38) | -2.03 (60) |
| DNA Replication | 1.92 (36) | --- | -2.1 (38) | -2.05 (43) |
| Establishment And Or Maintenance Of Chromatin Architecture | 1.66 (26) | --- | -1.80 (26) | -1.66 (24) |
| G Protein Coupled Receptor Binding | -1.76 (11) | --- | 1.79 (10) | --- |
| G1 S Transition Of Mitotic Cell Cycle | 1.85 (12) | --- | -2.10 (11) | -1.92 (18) |
| Humoral Immune Response | -1.99 (17) | --- | 2.05 (15) | 1.92 (13) |
| Immune Response | -2.16 (71) | --- | 2.47 (71) | 2.18 (78) |
| Immune System Process | -2.10 (94) | --- | 2.35 (88) | 2.11 (113) |
| Inflammatory Response | -1.92 (36) | --- | 1.81 (30) | 1.89 (36) |
| Interphase | 1.88 (24) | --- | -2.15 (21) | -2.07 (32) |
| Interphase Of Mitotic Cell Cycle | 1.88 (23) | --- | -2.21 (21) | -2.02 (34) |
| Leukocyte Activation | -1.96 (24) | --- | 2.16 (23) | 2.00 (27) |
| Lipid Raft | -1.72 (10) | --- | 1.94 (7) | 1.68 (8) |
| Lymphocyte Activation | -1.95 (23) | --- | 2.13 (21) | 1.95 (25) |
| M Phase | 1.98 (29) | --- | -2.33 (32) | -2.14 (39) |
| M Phase Of Mitotic Cell Cycle | 1.93 (37) | --- | -2.30 (38) | -2.04 (48) |
| Meiotic Cell Cycle | 1.59 (10) | --- | -1.92 (8) | -1.85 (11) |
| Microtubule | 1.83 (12) | --- | -2.01 (14) | -1.81 (13) |
| Microtubule Associated Complex | 1.59 (12) | --- | -1.71 (12) | --- |
| Microtubule Cytoskeleton | 1.78 (49) | --- | -2.16 (45) | -1.87 (44) |
| Microtubule Cytoskeleton Organization And Biogenesis | 1.64 (7) | --- | -2.12 (9) | -2.00 (10) |
| Microtubule Organizing Center | 1.69 (19) | --- | -2.03 (7) | -1.73 (24) |
| Microtubule Organizing Center Organization And Biogenesis | 1.70 (11) | --- | -1.91 (22) | -1.65 (15) |
| Mitochondrial Membrane Part | 1.73 (22) | --- | -1.88 (35) | -1.93 (26) |
| Mitosis | 1.94 (28) | --- | -2.25 (31) | -2.03 (36) |
| Mitotic Cell Cycle | 1.95 (50) | --- | -2.49 (51) | -2.19 (67) |
| mRNA Metabolic Process | 1.73 (25) | --- | -2.27 (30) | -1.93 (32) |
| mRNA Processing GO 0006397 | 1.84 (24) | --- | -2.26 (26) | -1.96 (29) |
| Neurite Development | -1.66 (7) | --- | 1.72 (9) | 1.68 (13) |
| Nuclear Envelope | 1.59 (25) | --- | -1.86 (29) | -1.83 (36) |
| Nuclear Lumen | 1.59 (110) | --- | -2.03 (105) | -1.87 (120) |
| Nuclear Membrane | 1.67 (20) | --- | -2.02 (24) | -2.01 (28) |
| Nuclear Membrane Part | 1.74 (22) | --- | -2.18 (26) | -2.06 (31) |
| Nuclear Part | 1.78 (157) | --- | -2.29 (194) | -2.06 (198) |
| Nuclear Pore | 1.74 (18) | --- | -2.04 (20) | -1.96 (24) |
| Nucleoplasm | 1.64 (71) | --- | -1.83 (66) | -1.76 (67) |
| Nucleoplasm Part | 1.60 (88) | --- | -1.80 (83) | -1.66 (87) |
| Pore Complex | 1.73 (19) | --- | -2.06 (23) | -2.03 (26) |
| Positive Regulation Of Cytokine Biosynthetic Process | -1.74 (10) | --- | 1.95 (12) | 1.76 (9) |
| Positive Regulation Of Immune System Process | -1.66 (14) | --- | 1.96 (18) | 1.71 (15) |
| Protein DNA Complex Assembly | 1.79 (25) | --- | -1.70 (17) | -1.68 (27) |
| Protein RNA Complex Assembly | 1.68 (18) | --- | -1.79 (23) | -1.92 (23) |
| Regulation Of Cell Cycle | 1.67 (48) | --- | -1.73 (51) | -1.79 (62) |
| Regulation Of Cytokine Biosynthetic Process | -1.77 (15) | --- | 1.82 (16) | 1.81 (12) |
| Regulation Of Immune System Process | -1.72 (19) | --- | 2.01 (23) | 1.77 (19) |
| Regulation Of Lymphocyte Activation | -1.72 (14) | --- | 1.99 (14) | 1.80 (15) |
| Regulation Of Mitosis | 1.67 (13) | --- | -1.63 (11) | -1.62 (16) |
| Regulation Of T Cell Activation | -1.65 (12) | --- | 1.94 (13) | 1.69 (11) |
| Response To DNA Damage Stimulus | 1.85 (61) | --- | -1.93 (44) | -1.93 (70) |
| Response To Endogenous Stimulus | 1.78 (67) | --- | -1.92 (56) | -1.88 (77) |
| Ribonucleoprotein Complex Biogenesis And Assembly | 1.69 (21) | --- | -2.04 (41) | -1.92 (48) |
| RNA Export From Nucleus | 1.61 (8) | --- | -1.76 (13) | -1.71 (13) |
| RNA Processing | 1.94 (56) | --- | -2.28 (54) | -2.11 (69) |
| RNA Splicing | 1.86 (30) | --- | -2.25 (26) | -2.03 (37) |
| RNA Splicingvia Transesterification Reactions | 1.64 (10) | --- | -2.05 (12) | -1.75 (11) |
| Spindle Pole | 1.84 (10) | --- | -2.10 (17) | -1.76 (20) |
| Spliceosome | 1.72 (15) | --- | -2.08 (17) | -1.88 (21) |
| T Cell Activation | -1.77 (17) | --- | 2.12 (18) | 1.80 (18) |
| Translation Factor Activity Nucleic Acid Binding | 1.89 (17) | --- | -1.92 (19) | -1.85 (17) |
| Translation Initiation Factor Activity | 1.81 (12) | --- | -1.73 (13) | -1.80 (11) |
| Translation Regulator Activity | 1.87 (17) | --- | -1.84 (19) | -1.86 (18) |
| tRNA Metabolic Process | 1.61 (7) | --- | -1.61 (10) | -1.73 (10) |
| Unfolded Protein Binding | 1.60 (19) | --- | -1.94 (18) | -1.78 (25) |
| Cytokine Binding | --- | 1.89 (19) | 1.81 (18) | 1.82 (20) |
| Nucleolus | --- | -1.85 (29) | -2.06 (39) | -1.88 (41) |
| Chromatin Assembly | 1.61 (10) | --- | -1.64 (7) | --- |
| Calcium Ion Transport | -1.68 (6) | --- | --- | 1.63 (10) |
| Caspase Activation | -1.66 (10) | --- | --- | 1.63 (10) |
| Generation Of Neurons | -1.71 (14) | --- | --- | 1.70 (19) |
| Hemopoietic Or Lymphoid Organ Development | -1.66 (22) | --- | --- | 1.59 (26) |
| Interleukin Receptor Activity | -1.70 (8) | --- | --- | 1.67 (12) |
| Ligase Activity | 1.79 (29) | --- | --- | -1.73 (27) |
| Ligase Activity Forming Carbon Nitrogen Bonds | 1.73 (41) | --- | --- | -1.60 (39) |
| Multi Organism Process | -1.67 (32) | --- | --- | 1.63 (29) |
| Neuron Differentiation | -1.72 (12) | --- | --- | 1.72 (18) |
| Nuclear Body | 1.71 (14) | --- | --- | -1.77 (15) |
| Nucleotide Biosynthetic Process | 1.62 (8) | --- | --- | -1.61 (7) |
| Nucleotide Metabolic Process | 1.66 (16) | --- | --- | -1.93 (17) |
| Oxidoreductase Activity Acting On NADH Or NADPH | 1.58 (12) | --- | --- | -1.61 (10) |
| Positive Regulation Of Caspase Activity | -1.70 (10) | --- | --- | 1.60 (10) |
| Protein Modification By Small Protein Conjugation | 1.64 (14) | --- | --- | -1.54 (20) |
| RAS Gtpase Activator Activity | -1.98 (13) | --- | --- | 1.67 (12) |
| Response To Wounding | -1.78 (47) | --- | --- | 1.85 (43) |
| RHO GTPase Activator Activity | -1.90 (8) | --- | --- | 1.76 (6) |
| Ubiquitin Cycle | 1.66 (15) | --- | --- | -1.63 (16) |
| Nucleobasenucleoside And Nucleotide Metabolic Process | --- | -1.88 (20) | --- | -2.01 (18) |
| Adherens Junction | -1.65 (8) | --- | --- | --- |
| Aerobic Respiration | 1.64 (7) | --- | --- | --- |
| Leukocyte Differentiation | -1.80 (13) | --- | --- | --- |
| Positive Regulation Of Hydrolase Activity | -1.71 (15) | --- | --- | --- |
| Protein Domain Specific Binding | -1.71 (21) | --- | --- | --- |
| Regulation Of Anatomical Structure Morphogenesis | -1.68 (6) | --- | --- | --- |
| Response To Temperature Stimulus | 1.73 (8) | --- | --- | --- |
| Thiolester Hydrolase Activity | 1.65 (7) | --- | --- | --- |
| Transcription Initiation | 1.63 (17) | --- | --- | --- |
| Transcription Initiation From RNA Polymerase Ii Promoter | 1.64 (18) | --- | --- | --- |
| Structural Constituent Of Muscle | --- | 1.86 (6) | --- | --- |
| Actin Cytoskeleton | --- | --- | 1.74 (27) | 1.78 (33) |
| Actin Cytoskeleton Organization And Biogenesis | --- | --- | 1.78 (27) | 1.76 (38) |
| Actin Filament Based Process | --- | --- | 1.82 (30) | 1.80 (37) |
| Actin Polymerization And Or Depolymerization | --- | --- | 1.81 (11) | 1.72 (10) |
| Adaptive Immune Response | --- | --- | 2.14 (14) | 1.64 (14) |
| Adaptive Immune Response GO 0002460 | --- | --- | 2.08 (15) | 1.62 (11) |
| Cell Projection | --- | --- | 1.86 (20) | 1.73 (28) |
| Chromatin | --- | --- | -1.83 (13) | -1.56 (12) |
| Chromatin Binding | --- | --- | -2.17 (6) | -1.88 (7) |
| Chromatin Remodeling Complex | --- | --- | -1.72 (6) | -1.55 (12) |
| Cofactor Biosynthetic Process | --- | --- | -1.73 (11) | -1.64 (16) |
| DNA Dependent ATPase Activity | --- | --- | -1.90 (11) | -1.67 (9) |
| Cofactor Metabolic Process | --- | --- | -1.79 (18) | -1.73 (24) |
| Condensed Nuclear Chromosome | --- | --- | -1.98 (11) | -1.82 (10) |
| Cytokine Activity | --- | --- | 1.80 (28) | 1.62 (24) |
| Envelope | --- | --- | -2.08 (68) | -1.87 (75) |
| Heterocycle Metabolic Process | --- | --- | -1.73 (11) | -1.64 (12) |
| Hydrolase Activity Hydrolyzing O Glycosyl Compounds | --- | --- | 1.71 (8) | 1.66 (13) |
| I Kappab Kinase NF Kappab Cascade | --- | --- | 1.85 (26) | 1.63 (34) |
| Kinetochore | --- | --- | -2.04 (13) | -1.72 (12) |
| Membrane Enclosed Lumen | --- | --- | -2.02 (126) | -1.89 (148) |
| Microtubule Binding | --- | --- | -1.59 (13) | -1.54 (14) |
| Mitochondrial Inner Membrane | --- | --- | -1.75 (23) | -1.84 (29) |
| Mitochondrial Lumen | --- | --- | -1.95 (21) | -1.76 (22) |
| Mitochondrial Matrix | --- | --- | -1.94 (21) | -1.78 (22) |
| Mitochondrial Part | --- | --- | -2.10 (61) | -1.75 (62) |
| Mitochondrial Ribosome | --- | --- | -2.07 (12) | -1.64 (13) |
| Mitochondrion | --- | --- | -2.12 (121) | -1.81 (123) |
| Neuron Development | --- | --- | 1.71 (11) | 1.68 (15) |
| Nuclear Chromosome | --- | --- | -2.09 (10) | -1.67 (17) |
| Nuclear Export | --- | --- | -1.72 (16) | -1.66 (19) |
| Nucleobasenucleosidenucleotide And Nucleic Acid Transport | --- | --- | -1.76 (18) | -1.80 (19) |
| Nucleotidyltransferase Activity | --- | --- | -1.62 (20) | -1.62 (20) |
| Organellar Ribosome | --- | --- | -2.12 (12) | -1.67 (13) |
| Organelle Envelope | --- | --- | -2.08 (68) | -1.84 (75) |
| Organelle Inner Membrane | --- | --- | -1.84 (26) | -1.88 (32) |
| Organelle Lumen | --- | --- | -2.03 (126) | -1.90 (148) |
| Peptidyl Tyrosine Modification | --- | --- | 1.94 (9) | 1.69 (11) |
| Peptidyl Tyrosine Phosphorylation | --- | --- | 1.88 (8) | 1.63 (9) |
| Positive Regulation Of Cellular Protein Metabolic Process | --- | --- | 1.75 (25) | 1.74 (23) |
| Positive Regulation Of I Kappab Kinase NF Kappab Cascade | --- | --- | 1.82 (22) | 1.61 (28) |
| Positive Regulation Of Lymphocyte Activation | --- | --- | 1.90 (11) | 1.72 (11) |
| Positive Regulation Of Protein Metabolic Process | --- | --- | 1.72 (25) | 1.72 (23) |
| Positive Regulation Of T Cell Activation | --- | --- | 1.83 (10) | 1.62 (9) |
| Positive Regulation Of Translation | --- | --- | 1.79 (14) | 1.62 (10) |
| Protein Folding | --- | --- | -1.91 (18) | -1.75 (27) |
| Receptor Binding | --- | --- | 1.90 (84) | 1.67 (68) |
| Regulation Of DNA Metabolic Process | --- | --- | -1.68 (8) | -1.58 (18) |
| Regulation Of I Kappab Kinase NF Kappab Cascade | --- | --- | 1.81 (23) | 1.60 (29) |
| Regulation Of Multicellular Organismal Process | --- | --- | 1.70 (36) | 1.61 (34) |
| Ribonucleoprotein Complex | --- | --- | -2.07 (29) | -1.97 (26) |
| Ribosomal Subunit | --- | --- | -1.97 (10) | -1.57 (11) |
| Ribosome | --- | --- | -1.87 (13) | -1.58 (15) |
| RNA Binding | --- | --- | -1.59 (57) | -1.73 (84) |
| Small Nuclear Ribonucleoprotein Complex | --- | --- | -1.99 (8) | -1.66 (9) |
| Spindle | --- | --- | -2.08 (9) | -1.89 (9) |
| Structure Specific DNA Binding | --- | --- | -1.60 (13) | -1.72 (25) |
| Antioxidant Activity | --- | --- | -1.64 (8) | --- |
| ATP Dependent Helicase Activity | --- | --- | -1.69 (10) | --- |
| Behavior | --- | --- | 1.92 (39) | --- |
| Cation Homeostasis | --- | --- | 1.82 (20) | --- |
| Cell Division | --- | --- | -1.87 (6) | --- |
| Cellular Cation Homeostasis | --- | --- | 1.88 (20) | --- |
| Chemical Homeostasis | --- | --- | 1.73 (26) | --- |
| Cytoskeletal Part | --- | --- | -1.75 (54) | --- |
| Cytosolic Part | --- | --- | -1.67 (6) | --- |
| DNA Directed RNA Polymeraseii Holoenzyme | --- | --- | -1.68 (18) | --- |
| DNA Helicase Activity | --- | --- | -1.87 (11) | --- |
| Drug Binding | --- | --- | -1.81 (7) | --- |
| Endonuclease Activity | --- | --- | -1.68 (9) | --- |
| Exonuclease Activity | --- | --- | -1.72 (5) | --- |
| Glucose Metabolic Process | --- | --- | -1.59 (8) | --- |
| Helicase Activity | --- | --- | -1.72 (16) | --- |
| Hydrolase Activity Acting On Carbon Nitrogen Not Peptidebonds | --- | --- | -1.82 (10) | --- |
| Ion Homeostasis | --- | --- | 1.70 (24) | --- |
| Isomerase Activity | --- | --- | -1.83 (12) | --- |
| Lamellipodium | --- | --- | 1.93 (8) | --- |
| Leading Edge | --- | --- | 1.74 (13) | --- |
| Locomotory Behavior | --- | --- | 1.85 (21) | --- |
| Microtubule Organizing Center Part | --- | --- | -1.68 (12) | --- |
| Mitochondrial Envelope | --- | --- | -1.83 (40) | --- |
| Mitochondrial Membrane | --- | --- | -1.75 (25) | --- |
| Nuclear Chromosome Part | --- | --- | -1.74 (19) | --- |
| Nuclear Transport | --- | --- | -1.59 (35) | --- |
| Nucleocytoplasmic Transport | --- | --- | -1.61 (35) | --- |
| Nucleolar Part | --- | --- | -1.76 (7) | --- |
| Organelle Membrane | --- | --- | -1.58 (89) | --- |
| Pigment Biosynthetic Process | --- | --- | -1.69 (8) | --- |
| Pigment Metabolic Process | --- | --- | -1.66 (8) | --- |
| Regulation Of Mitotic Cell Cycle | --- | --- | -1.74 (5) | --- |
| Regulation Of Protein Stability | --- | --- | -1.59 (4) | --- |
| Replication Fork | --- | --- | -1.65 (9) | --- |
| Ribonuclease Activity | --- | --- | -1.74 (11) | --- |
| RNA Polymerase Activity | --- | --- | -1.84 (7) | --- |
| Transition Metal Ion Binding | --- | --- | -1.59 (23) | --- |
| Acetyltransferase Activity | --- | --- | --- | -1.68 (6) |
| Actin Binding | --- | --- | --- | 1.59 (27) |
| Anion Channel Activity | --- | --- | --- | -1.62 (2) |
| Cellular Protein Catabolic Process | --- | --- | --- | -1.60 (28) |
| Chloride Channel Activity | --- | --- | --- | -1.58 (2) |
| Chromatin Remodeling | --- | --- | --- | -1.53 (8) |
| Development Of Primary Sexual Characteristics | --- | --- | --- | -1.56 (11) |
| DNA Integrity Checkpoint | --- | --- | --- | -1.56 (10) |
| Double Strand Break Repair | --- | --- | --- | -1.66 (10) |
| Double Stranded DNA Binding | --- | --- | --- | -1.76 (16) |
| Hemopoiesis | --- | --- | --- | 1.59 (25) |
| Meiosis I | --- | --- | --- | -1.54 (6) |
| Methyltransferase Activity | --- | --- | --- | -1.60 (19) |
| Mitochondrial Respiratory Chain | --- | --- | --- | -1.54 (11) |
| Muscle Development | --- | --- | --- | 1.63 (20) |
| N Acyltransferase Activity | --- | --- | --- | -1.72 (7) |
| Positive Regulation Of Cell Differentiation | --- | --- | --- | 1.59 (11) |
| Positive Regulation Of Phosphate Metabolic Process | --- | --- | --- | 1.73 (11) |
| Positive Regulation Of Phosphorylation | --- | --- | --- | 1.77 (10) |
| Positive Regulation Of Protein Amino Acid Phosphorylation | --- | --- | --- | 1.72 (9) |
| Positive Regulation Of Protein Modification Process | --- | --- | --- | 1.60 (12) |
| Protease Inhibitor Activity | --- | --- | --- | 1.59 (8) |
| Proteasome Complex | --- | --- | --- | -1.57 (11) |
| Protein Catabolic Process | --- | --- | --- | -1.54 (30) |
| RAS Protein Signal Transduction | --- | --- | --- | 1.66 (22) |
| Receptor Signaling Protein Activity | --- | --- | --- | 1.71 (22) |
| Regulation Of Cell Differentiation | --- | --- | --- | 1.69 (17) |
| Regulation Of Cyclin Dependent Protein Kinase Activity | --- | --- | --- | -1.64 (19) |
| Regulation Of Protein Amino Acid Phosphorylation | --- | --- | --- | 1.70 (10) |
| Regulation Of Protein Import Into Nucleus | --- | --- | --- | 1.62 (5) |
| Response To ExteRNAl Stimulus | --- | --- | --- | 1.71 (63) |
| Single Stranded DNA Binding | --- | --- | --- | -1.52 (16) |
| Skeletal Development | --- | --- | --- | 1.70 (15) |
| Small GTPase Mediated Signal Transduction | --- | --- | --- | 1.64 (24) |
| Small GTPase Regulator Activity | --- | --- | --- | 1.62 (21) |
| Transferase Activity Transferring Groups Other Than Amino Acyl Groups | --- | --- | --- | -1.61 (14) |
| Transferase Activity Transferring One Carbon Groups | --- | --- | --- | -1.56 (19) |
| Translational Initiation | --- | --- | --- | -1.70 (13) |
| Tubulin Binding | --- | --- | --- | -1.54 (14) |

**Supplemental Table 1.** Gene Set Enrichment Analysis (GSEA) for GO gene sets in the spleen with different age and proton irradiation comparisons (0Gy Adolescent (A), 0Gy Old (O), 0.5Gyx3 Proton Adolescent (A P), 0.5Gyx3 Proton Old (O P)). Leading edge analysis with a FDR < 0.05 determined significant gene sets enriched for each group. The normalized enrichment score (NES) indicates whether the gene set is up- (>0) or down-regulated (<0) for each group determined with the # of leading edge genes (appears in parenthesis).
