# Supplementary material for: Proton irradiation impacts age-driven modulations of cancer progression influenced by immune system transcriptome modifications from splenic tissue
Source: J Radiat Res. 2015 Aug 7;56(5):792–803. doi: 10.1093/jrr/rrv043 (PMC4577010; doi:10.1093/jrr/rrv043)
Supplement: Supplementary Data [file supp_rrv043_rrv043supp_table2.doc]

| **Category** | **Functions** | **Diseases or Functions Annotation** | **Activation z-score (# of Genes)** | | | |
| --- | --- | --- | --- | --- | --- | --- |
|  |  |  | **A P vs A** | **O P vs O** | **O vs A** | **O P vs A P** |
| Cellular Movement; Immune Cell Trafficking; Inflammatory Response; & Hematological System Development and Function | chemotaxis | chemotaxis of phagocytes | -2.922 (37) | 2.461 (25) | 2.899 (12) | 3.574 (37) |
| Cellular Movement; Immune Cell Trafficking; Inflammatory Response; & Hematological System Development and Function | chemotaxis | chemotaxis of leukocytes | -3.381 (39) | 2.508 (31) | 2.516 (15) | --- |
| Cellular Movement; Immune Cell Trafficking; & Hematological System Development and Function | cell movement | cell movement of leukocytes | -3.459 (90) | 2.05 (62) | --- | 3.241 (101) |
| Cellular Movement; Immune Cell Trafficking; & Hematological System Development and Function | cell movement | cell movement of lymphocytes | -2.811 (44) | 2.116 (29) | --- | 2.901 (47) |
| Cellular Movement; Immune Cell Trafficking; & Hematological System Development and Function | homing | homing of leukocytes | -3.384 (43) | 2.686 (32) | --- | 3.923 (46) |
| Cell Cycle | interphase | interphase | 2.675 (88) | -2.250 (59) | --- | -2.954 (96) |
| Cellular Movement | migration | migration of cells | -2.279 (189) | 2.818 (125) | --- | 2.477 (209) |
| Cellular Movement; Immune Cell Trafficking | migration | leukocyte migration | -3.232 (103) | 2.18 (69) | --- | 3.125 (114) |
| Cellular Movement; Immune Cell Trafficking; & Hematological System Development and Function | migration | Lymphocyte migration | -2.273 (38) | 2.204 (25) | --- | 2.429 (41) |
| Cellular Movement; Immune Cell Trafficking; Inflammatory Response; & Hematological System Development and Function | chemotaxis | chemotaxis of myeloid cells | -2.768 (34) | 2.204 (25) | --- | --- |
| Cellular Development; Hematopoiesis; Hematological System Development and Function | development | development of blood cells | -2.662 (73) | --- | 2.015 (25) | 2.363 (88) |
| Tissue Morphology; Hematological System Development and Function | quantity | quantity of lymphocytes | -2.735 (83) | --- | 2.404 (33) | 2.566 (98) |
| Tissue Morphology; Hematological System Development and Function | quantity | quantity of mononuclear leukocytes | -2.472 (85) | --- | 2.169 (34) | 2.333 (102) |
| Cell-To-Cell Signaling and Interaction | response | response of antigen presenting cells | -3.187 (22) | --- | 2.177 (8) | 2.541 (24) |
| Tissue Morphology | quantity | quantity of cells | -2.476 (179) | --- | 2.362 (61) | --- |
| Tissue Morphology; Hematological System Development and Function | quantity | quantity of leukocytes | -2.085 (108) | --- | 2.014 (40) | --- |
| Cellular Movement; Immune Cell Trafficking; & Hematological System Development and Function | cell movement | cell movement of mononuclear leukocytes | -3.220 (54) | --- | --- | 3.066 (59) |
| Cellular Movement; Immune Cell Trafficking; & Hematological System Development and Function | cell movement | cell movement of myeloid cells | -2.557 (59) | --- | --- | 2.495 (68) |
| Cell Cycle; DNA Replication, Recombination, and Repair | checkpoint control | checkpoint control | 2.425 (21) | --- | --- | -2.941 (24) |
| Cellular Development; Hematopoiesis; Lymphoid Tissue Structure and Development; Hematological System Development and Function | development | development of leukocytes | -2.697 (65) | --- | --- | 2.403 (78) |
| Cellular Development; Hematopoiesis; Lymphoid Tissue Structure and Development; Hematological System Development and Function | development | development of lymphocytes | -2.413 (62) | --- | --- | 2.28 (76) |
| Hematopoiesis; Cell-mediated Immune Response; Cellular Development; Lymphoid Tissue Structure and Development; Cellular Function and Maintenance; Hematological System Development and Function | development | T cell development | -2.212 (56) | --- | --- | 2.067 (66) |
| Cell Cycle | G1 phase | G1 phase | 2.343 (54) | --- | --- | -2.343 (55) |
| Cell Cycle | G1/S phase transition | G1/S phase | 2.891 (24) | --- | --- | -2.077 (28) |
| Infectious Disease | HIV infection | HIV infection | 3.253 (87) | --- | --- | -3.825 (89) |
| Cellular Function and Maintenance | homeostasis | homeostasis of leukocytes | -2.488 (64) | --- | --- | 2.38 (74) |
| Cellular Function and Maintenance | homeostasis | Lymphocyte homeostasis | -2.488 (61) | --- | --- | 2.38 (72) |
| Cellular Function and Maintenance; Hematological System Development and Function | homeostasis | T cell homeostasis | -2.328 (59) | --- | --- | 2.18 (69) |
| Cellular Movement | homing | homing of blood cells | -3.384 (44) | --- | --- | 4.035 (48) |
| Cellular Movement | homing | homing of cells | -2.956 (60) | --- | --- | 3.343 (66) |
| Inflammatory Response | immune response | immune response of cells | -2.585 (36) | --- | --- | 3.039 (39) |
| Infectious Disease | infection | infection by HIV-1 | 3.279 (80) | --- | --- | -3.855 (82) |
| Infectious Disease | infection | infection by Retroviridae | 3.356 (89) | --- | --- | -3.909 (91) |
| Infectious Disease | infection | infection of cells | 3.577 (102) | --- | --- | -4.425 (103) |
| Infectious Disease; Organismal Injury and Abnormalities | infection | infection of embryonic cell lines; infection of epithelial cell lines | 2.052 (42) | --- | --- | -3.397 (39) |
| Infectious Disease; Reproductive System Disease | infection | infection of cervical cancer cell lines | 3.418 (54) | --- | --- | -2.705 (57) |
| Cell Cycle | interphase | interphase of bone cancer cell lines | 2.200 (12) | --- | --- | -2.200 (13) |
| Cell Cycle | interphase | interphase of tumor cell lines | 3.572 (45) | --- | --- | -3.363 (46) |
| Cellular Movement; Immune Cell Trafficking; & Hematological System Development and Function | migration | migration of mononuclear leukocytes | -2.557 (41) | --- | --- | 2.575 (45) |
| Cellular Development; Cellular Growth and Proliferation | proliferation | proliferation of blood cells | -2.279 (96) | --- | --- | 2.620 (113) |
| Cellular Development; Cellular Growth and Proliferation; Hematological System Development and Function | proliferation | proliferation of mononuclear leukocytes | -2.497 (82) | --- | --- | 2.531 (97) |
| Cellular Development; Cellular Growth and Proliferation; Hematological System Development and Function | proliferation | proliferation of immune cells | -2.482 (89) | --- | --- | 2.576 (106) |
| Cellular Development; Cellular Growth and Proliferation; Hematological System Development and Function | proliferation | proliferation of lymphocytes | -2.394 (79) | --- | --- | 2.551 (95) |
| Tissue Morphology; Hematological System Development and Function | quantity | quantity of T lymphocytes | -2.314 (61) | --- | --- | 2.075 (70) |
| Cell Cycle | S phase | S phase of bone cancer cell lines | 2.200 (5) | --- | --- | -2.200 (5) |
| Embryonic Development | size | size of embryo | 2.501 (54) | --- | --- | -3.614 (52) |
| Cell Death and Survival | cell viability | cell viability of blood cells | -2.437 (32) | --- | --- | --- |
| Cell Death and Survival | cell viability | cell viability of mononuclear leukocytes | -2.213 (24) | --- | --- | --- |
| Cellular Movement; Immune Cell Trafficking; Inflammatory Response; & Hematological System Development and Function | chemotaxis | chemotaxis of macrophages | -2.178 (14) | --- | --- | --- |
| Cellular Development; Hematopoiesis; Lymphoid Tissue Structure and Development; Hematological System Development and Function | development | development of mononuclear leukocytes | -2.563 (63) | --- | --- | --- |
| Hematopoiesis; Cellular Development; Hematological System Development and Function | differentiation | differentiation of mononuclear leukocytes | -2.23 (58) | --- | --- | --- |
| Hematopoiesis; Cellular Development; Hematological System Development and Function | differentiation | differentiation of lymphocytes | -2.115 (55) | --- | --- | --- |
| Infectious Disease | infection | Viral Infection | 3.201 (188) | --- | --- | --- |
| Infectious Disease | infection | infection of tumor cell lines | 3.231 (59) | --- | --- | --- |
| DNA Replication, Recombination, and Repair | metabolism | metabolism of DNA | 2.867 (66) | --- | --- | --- |
| Cellular Movement; Immune Cell Trafficking; Inflammatory Response; & Hematological System Development and Function | migration | migration of macrophages | -2.126 (12) | --- | --- | --- |
| Cancer; Gastrointestinal Disease | neoplasia | neoplasia of stomach | -2.000 (33) | --- | --- | --- |
| Cancer; Gastrointestinal Disease; Endocrine System Disorders | pancreatic tumor | pancreatic tumor | -2.387 (37) | --- | --- | --- |
| Infectious Disease; Reproductive System Disease | productive infection | productive infection of cervical cancer cell lines | 2.268 (29) | --- | --- | --- |
| Cellular Development; Cellular Growth and Proliferation; Hematological System Development and Function | proliferation | proliferation of T lymphocytes | -2.632 (64) | --- | --- | --- |
| Protein Synthesis; Humoral Immune Response | quantity | quantity of immunoglobulin | 2.063 (39) | --- | --- | --- |
| DNA Replication, Recombination, and Repair | replication | DNA replication | 2.851 (44) | --- | --- | --- |
| Cellular Movement; Immune Cell Trafficking; Inflammatory Response; & Hematological System Development and Function | cell movement | cell movement of phagocytes | --- | 2.185 (42) | 2.104 (20) | 3.116 (69) |
| Tissue Morphology; Humoral Immune Response; Lymphoid Tissue Structure and Development; Hematological System Development and Function | quantity | quantity of follicular B lymphocytes | --- | 2.429 (11) | 2.112 (10) | 2.887 (20) |
| Cellular Movement; Immune Cell Trafficking; & Hematological System Development and Function | cell movement | cell movement of granulocytes | --- | 2.276 (30) | --- | 2.565 (46) |
| Cell Death and Survival | apoptosis | apoptosis of breast cancer cell lines | --- | 2.462 (21) | --- | --- |
| Cell Death and Survival | cell death | cell death of breast cancer cell lines | --- | 2.122 (28) | --- | --- |
| Cellular Movement | cell movement | cell movement of tumor cell lines | --- | 2.739 (53) | --- | --- |
| Cellular Movement | cell movement | cell movement | --- | 2.466 (131) | --- | --- |
| Cellular Movement; Immune Cell Trafficking; & Hematological System Development and Function | cell movement | cell movement of neutrophils | --- | 3.109 (25) | --- | --- |
| Inflammatory Response | cell movement | cell movement of neutrophils | --- | 3.109 (25) | --- | --- |
| Cell Death and Survival | cell viability | cell viability of tumor cell lines | --- | -2.159 (59) | --- | --- |
| Cell Death and Survival; Hematological System Development and Function | cell viability | cell viability of T lymphocytes | --- | -2.061 (11) | --- | --- |
| Cellular Movement | chemotaxis | chemotaxis of cells | --- | 2.33 (40) | --- | --- |
| Cellular Movement; Immune Cell Trafficking; Inflammatory Response; & Hematological System Development and Function | chemotaxis | chemotaxis of neutrophils | --- | 2.548 (18) | --- | --- |
| Cellular Movement | homing | homing of cells | --- | 2.197 (44) | --- | --- |
| Cellular Movement; Immune Cell Trafficking; & Hematological System Development and Function | homing | homing of lymphocytes | --- | 2.052 (12) | --- | --- |
| Cellular Movement; Immune Cell Trafficking; Humoral Immune Response; & Hematological System Development and Function | homing | homing of B lymphocytes | --- | 2.164 (5) | --- | --- |
| Cellular Movement; Immune Cell Trafficking; Inflammatory Response; & Hematological System Development and Function | homing | homing of neutrophils | --- | 2.548 (19) | --- | --- |
| Cancer; Tumor Morphology | invasion | invasion of tumor | --- | -2.449 (10) | --- | --- |
| Cellular Movement | migration | migration of tumor cell lines | --- | 2.129 (44) | --- | --- |
| Tissue Morphology; Inflammatory Response; Hypersensitivity Response; Hematological System Development and Function | quantity | quantity of mast cells | --- | 2.050 (7) | --- | --- |
| Cell Morphology | morphology | morphology of mononuclear leukocytes | --- | --- | -2.608 (15) | -2.260 (34) |
| Cell Morphology | morphology | morphology of lymphocytes | --- | --- | -2.608 (14) | -2.260 (32) |
| Inflammatory Response | inflammatory response | inflammatory response | --- | --- | 2.645 (28) | 2.655 (82) |
| Cellular Development; Cellular Growth and Proliferation; Humoral Immune Response; Hematological System Development and Function | proliferation | proliferation of B lymphocytes | --- | --- | 2.571 (15) | 2.257 (41) |
| Inflammatory Response & Antigen Presentation | antigen presentation | antigen presentation | --- | --- | 2.200 (8) | --- |
| Cell Death and Survival | cell death | cell death of colon cancer cell lines | --- | --- | 2.295 (9) | --- |
| Cellular Movement; Immune Cell Trafficking; Inflammatory Response; & Hematological System Development and Function | cell movement | cell movement of dendritic cells | --- | --- | 2.163 (8) | --- |
| Cellular Movement; Immune Cell Trafficking; Inflammatory Response; & Hematological System Development and Function | chemotaxis | chemotaxis of antigen presenting cells | --- | --- | 2.145 (8) | --- |
| Cellular Compromise; Cell Death and Survival | cytotoxicity | cytotoxicity of cells | --- | --- | 2.396 (10) | --- |
| Cellular Compromise; Cell Death and Survival | cytotoxicity | cytotoxicity of leukocytes | --- | --- | 2.18 (8) | --- |
| Cellular Development; Hematological System Development and Function | differentiation | differentiation of blood cells | --- | --- | 2.511 (26) | --- |
| Hematopoiesis; Cellular Development; Hematological System Development and Function | differentiation | differentiation of hematopoietic progenitor cells | --- | --- | 2.365 (13) | --- |
| Cellular Growth and Proliferation; Hematological System Development and Function | inhibition | inhibition of lymphocytes | --- | --- | 2.099 (5) | --- |
| Cancer | lymphohematopoietic cancer | lymphohematopoietic cancer | --- | --- | -2.083 (33) | --- |
| Cell Morphology | morphology | morphology of blood cells | --- | --- | -2.18 (27) | --- |
| Cell Morphology | morphology | morphology of leukocytes | --- | --- | -2.18 (23) | --- |
| Humoral Immune Response; Protein Synthesis | quantity | quantity of IgG3 | --- | --- | 2.183 (5) | --- |
| Tissue Morphology; Hematological System Development and Function | quantity | quantity of blood cells | --- | --- | 2.277 (45) | --- |
| Cell Death and Survival | cell death | cell death of cervical cancer cell lines | --- | --- | --- | 2.142 (45) |
| Cellular Movement | cell movement | cell movement of blood cells | --- | --- | --- | 3.209 (115) |
| Cell Cycle; Cellular Assembly and Organization; DNA Replication, Recombination, and Repair | formation | formation of mitotic spindle | --- | --- | --- | 2.059 (12) |
| Cell Cycle; Cellular Assembly and Organization; DNA Replication, Recombination, and Repair | formation | formation of centriole | --- | --- | --- | -2.207 (6) |
| Cellular Assembly and Organization; DNA Replication, Recombination, and Repair | formation | formation of spindle fibers | --- | --- | --- | 2.274 (13) |
| Organismal Development | growth | growth of organism | --- | --- | --- | -2.208 (63) |
| Developmental Disorder | growth failure | Growth Failure | --- | --- | --- | 2.476 (76) |
| Infectious Disease | infection | infection of mammalia | --- | --- | --- | -2.573 (44) |
| Infectious Disease | infection | Viral Infection | --- | --- | --- | -3.332 (210) |
| Infectious Disease; Renal and Urological Disease | infection | infection of kidney cell lines | --- | --- | --- | -3.256 (40) |
| Cell Cycle | mitotic index | mitotic index | --- | --- | --- | 2.433 (6) |
| Embryonic Development; Organismal Survival | organismal death | death of embryo | --- | --- | --- | 3.054 (20) |
| Infectious Disease | replication | replication of RNA virus | --- | --- | --- | -2.001 (63) |
| Infectious Disease | replication | replication of virus | --- | --- | --- | -2.055 (71) |
| Cell Cycle; DNA Replication, Recombination, and Repair | S phase checkpoint control | S phase checkpoint control | --- | --- | --- | -2.190 (7) |

**Supplemental Table 2.** Biofunctions predicted to be activated or inhibited in the spleen with different age and proton irradiation comparisons (0Gy Adolescent (A), 0Gy Old (O), 0.5Gyx3 Proton Adolescent (A P), 0.5Gyx3 Proton Old (O P)) obtained through the use of Ingenuity Pathway Analysis (IPA) software. The predicted activation state of each of these functions is predicted to be increased (z-score ≥ 2) or decrease (z-score ≤ -2) by the use of the regulation z-score which indicates the degree of biofunction activity. The values in parenthesis indicate the number of genes IPA used in the prediction.
