# Supplementary material for: Proton irradiation impacts age-driven modulations of cancer progression influenced by immune system transcriptome modifications from splenic tissue
Source: J Radiat Res. 2015 Aug 7;56(5):792–803. doi: 10.1093/jrr/rrv043 (PMC4577010; doi:10.1093/jrr/rrv043)
Supplement: Supplementary Data [file supp_rrv043_rrv043supp_table3.doc]

| **Upstream Regulator** | **Molecule Type** | **Tumor Effect (Ref.)** | **Activation z-score (# of Genes)** | | | |
| --- | --- | --- | --- | --- | --- | --- |
|  |  |  | **A P vs A** | **O P vs O** | **O vs A** | **O P vs A P** |
| IFNA2 | cytokine | Inhibits | -2.084 (10) | 2.430 (9) | 2.234 (5) | 2.962 (15) |
| Ifnar | group | Inhibits | -2.287 (10) | 3.266 (11) | 3.568 (13) | 3.119 (15) |
| IFNG | cytokine | Inhibits | -3.347 (94) | 2.550 (64) | 4.584 (41) | 4.451 (107) |
| MYC | transcription regulator | Promotes | 3.662 (91) | -3.983 (59) | -3.784 (25) | -6.482 (111) |
| CDKN2A | transcription regulator | Inhibits | -3.937 (28) | 2.331 (13) | --- | 5.047 (36) |
| Rb | group | Inhibits | -2.377 (12) | 2.219 (7) | --- | 3.121 (14) |
| TBX2 | transcription regulator | Promotes | 2.138 (14) | -2.121 (8) | --- | -3.448 (19) |
| miR-155-5p | mature microRNA | Promotes | 2.358 (17) | 2.869 (15) | --- | --- |
| F2 | peptidase | Promotes | -2.561 (20) | --- | 2.560 (7) | 2.235 (23) |
| BNIP3L | other | Inhibits | -2.891 (12) | --- | 2.449 (6) | 3.860 (15) |
| IL6 | cytokine | Promotes | -3.152 (58) | --- | 2.737 (29) | 2.728 (65) |
| Interferon alpha | group | Inhibits | -2.206 (21) | --- | 2.502 (13) | 2.545 (24) |
| KDM5B | transcription regulator | Promotes | -3.060 (24) | --- | 2.420 (10) | 4.004 (24) |
| NUPR1 | transcription regulator | Promotes | -3.244 (47) | --- | 2.333 (10) | 3.713 (62) |
| RB1 | transcription regulator | Inhibits | -3.091 (30) | --- | 2.591 (10) | 3.368 (38) |
| RBL1 | transcription regulator | Inhibits | -2.874 (15) | --- | 2.236 (6) | 3.035 (17) |
| TNF | cytokine | Both | -2.613 (122) | --- | 2.745 (50) | 3.097 (142) |
| TP53 | transcription regulator | Inhibits | -3.200 (141) | --- | 3.160 (41) | 4.226 (167) |
| INSIG1 | other | Promotes | 2.121 (8) | --- | -2.000 (4) | --- |
| PRL | cytokine | Promotes | -2.294 (22) | --- | 2.128 (12) | --- |
| 26s Proteasome | complex | Both | 2.200 (5) | --- | --- | -2.200 (5) |
| Akt | group | Promotes | -2.049 (15) | --- | --- | 2.526 (15) |
| CD24 | other | Promotes | 2.236 (8) | --- | --- | -2.121 (13) |
| E2f | group | Promotes | 2.158 (18) | --- | --- | -2.961 (21) |
| E2F1 | transcription regulator | Inhibits | 2.948 (53) | --- | --- | -3.199 (63) |
| EP400 | other | Promotes | 2.121 (8) | --- | --- | -2.985 (9) |
| EPO | cytokine | Both | 2.103 (31) | --- | --- | -2.389 (34) |
| ERG | transcription regulator | Promotes | -2.121 (12) | --- | --- | 2.121 (12) |
| ERK1/2 | group | Promotes | -2.179 (8) | --- | --- | 2.286 (12) |
| ESRRA | ligand-dependent nuclear receptor | Promotes | 2.177 (8) | --- | --- | -2.390 (9) |
| HMGN5 | transcription regulator | Promotes | 2.236 (7) | --- | --- | -2.000 (6) |
| IKBKB | kinase | Promotes | -2.522 (25) | --- | --- | 2.469 (29) |
| IL1A | cytokine | Promotes | -2.920 (11) | --- | --- | 2.229 (12) |
| KLF2 | transcription regulator | Inhibits | -2.414 (15) | --- | --- | 2.414 (16) |
| NFE2L2 | transcription regulator | Promotes | 2.269 (38) | --- | --- | -2.702 (35) |
| NFYA | transcription regulator | Promotes | -2.000 (5) | --- | --- | 2.236 (6) |
| NLRP3 | other | Promotes | -2.236 (5) | --- | --- | 2.449 (6) |
| Notch | group | Promotes | -2.200 (5) | --- | --- | 2.191 (5) |
| PRNP | other | Promotes | -2.190 (5) | --- | --- | 2.190 (6) |
| PTEN | phosphatase | Inhibits | -2.403 (41) | --- | --- | 2.700 (45) |
| SMARCA4 | transcription regulator | Inhibits | -2.249 (35) | --- | --- | 2.621 (33) |
| STAT3 | transcription regulator | Promotes | -3.198 (25) | --- | --- | 2.258 (28) |
| TCF3 | transcription regulator | Promotes | -2.887 (28) | --- | --- | 4.123 (34) |
| TCR | complex | Inhibits | -2.130 (28) | --- | --- | 2.176 (35) |
| TGFA | growth factor | Promotes | -2.553 (7) | --- | --- | 2.362 (6) |
| TGFB1 | growth factor | Promotes | -3.092 (123) | --- | --- | 3.212 (147) |
| TGFBR2 | kinase | Inhibits | -2.768 (13) | --- | --- | 2.938 (15) |
| Tlr | group | Both | -2.000 (4) | --- | --- | 2.219 (5) |
| TLR3 | transmembrane receptor | Inhibits | -2.213 (6) | --- | --- | 2.213 (6) |
| TLR9 | transmembrane receptor | Inhibits | -2.216 (7) | --- | --- | 2.216 (7) |
| ARHGAP21 | other | Both | -2.236 (5) | --- | --- | --- |
| CD44 | enzyme | Both | -2.779 (14) | --- | --- | --- |
| COL18A1 | other | Inhibits | 2.343 (9) | --- | --- | --- |
| CSF3 | cytokine | Promotes | -2.288 (15) | --- | --- | --- |
| CXCL12 | cytokine | Promotes | -2.451 (13) | --- | --- | --- |
| DOCK8 | other | Inhibits | -2.000 (4) | --- | --- | --- |
| FGF1 | growth factor | Promotes | -2.364 (7) | --- | --- | --- |
| GATA4 | transcription regulator | Both | 2.000 (5) | --- | --- | --- |
| HBB | transporter | Inhibits | -2.000 (5) | --- | --- | --- |
| Hbb-b2 | other | N.D. | -2.000 (5) | --- | --- | --- |
| HIF1A | transcription regulator | Promotes | -2.564 (29) | --- | --- | --- |
| IL1 | group | Promotes | -2.353 (16) | --- | --- | --- |
| Jnk | group | Both | -2.055 (9) | --- | --- | --- |
| LDL | complex | Promotes | -2.054 (18) | --- | --- | --- |
| Map4k4 | kinase | Promotes | -2.309 (12) | --- | --- | --- |
| MARK2 | kinase | Inhibits | -2.000 (4) | --- | --- | --- |
| MAX | transcription regulator | Inhibits | 2.236 (9) | --- | --- | --- |
| NR1I2 | ligand-dependent nuclear receptor | Inhibits | 2.236 (6) | --- | --- | --- |
| RAF1 | kinase | Promotes | -2.052 (11) | --- | --- | --- |
| SASH1 | other | Inhibits | -2.000 (4) | --- | --- | --- |
| SELP | transmembrane receptor | Promotes | -2.000 (4) | --- | --- | --- |
| SELPLG | other | Inhibits | -2.000 (4) | --- | --- | --- |
| SPI1 | transcription regulator | Inhibits | -2.247 (17) | --- | --- | --- |
| TBX5 | transcription regulator | Inhibits | 2.000 (4) | --- | --- | --- |
| TP73 | transcription regulator | Inhibits | -2.252 (18) | --- | --- | --- |
| SPARC | other | Inhibits | --- | 2.714 (11) | 2.000 (4) | 2.458 (17) |
| STAT1 | transcription regulator | Promotes | --- | 2.132 (13) | 2.475 (18) | 2.133 (30) |
| CDKN1A | kinase | Inhibits | --- | 2.274 (21) | --- | 2.010 (47) |
| CSF2 | cytokine | Promotes | --- | -2.162 (33) | --- | -2.585 (57) |
| MKL1 | transcription regulator | Both | --- | 2.596 (11) | --- | 2.289 (16) |
| MYCN | transcription regulator | Promotes | --- | -3.364 (16) | --- | -3.296 (29) |
| POR | enzyme | Inhibits | --- | -2.425 (10) | --- | -2.213 (10) |
| SRF | transcription regulator | Promotes | --- | 2.599 (13) | --- | 2.148 (25) |
| AGT | growth factor | Promotes | --- | -2.032 (23) | --- | --- |
| EGR1 | transcription regulator | Promotes | --- | 2.213 (6) | --- | --- |
| ERK | group | Promotes | --- | -2.219 (6) | --- | --- |
| IL5 | cytokine | Promotes | --- | -2.000 (16) | --- | --- |
| IRF1 | transcription regulator | Inhibits | --- | 2.181 (14) | --- | --- |
| ITGB1 | transmembrane receptor | Promotes | --- | 2.219 (5) | --- | --- |
| Lh | complex | Promotes | --- | -2.000 (16) | --- | --- |
| MAPK9 | kinase | Both | --- | -2.200 (5) | --- | --- |
| Mek | group | Promotes | --- | -2.222 (6) | --- | --- |
| miR-16-5p | mature microRNA | Promotes | --- | 2.592 (11) | --- | --- |
| NKX2-3 | transcription regulator | Inhibits | --- | -2.828 (8) | --- | --- |
| PDGF BB | complex | Promotes | --- | -2.487 (21) | --- | --- |
| TNFSF10 | cytokine | Both | --- | 2.177 (5) | --- | --- |
| TREM1 | transmembrane receptor | Promotes | --- | -2.043 (8) | --- | --- |
| IL27 | cytokine | Inhibits | --- | --- | 2.600 (7) | 2.092 (10) |
| IRF7 | transcription regulator | Inhibits | --- | --- | 2.596 (7) | 2.026 (13) |
| let-7 | microRNA | Inhibits | --- | --- | 2.383 (11) | 3.153 (37) |
| NLRC5 | transcription regulator | Inhibits | --- | --- | 2.405 (6) | 2.405 (6) |
| TGM2 | enzyme | Promotes | --- | --- | 2.236 (5) | 2.000 (16) |
| Vegf | group | Promotes | --- | --- | -2.029 (18) | -2.022 (47) |
| Cg | complex | Promotes | --- | --- | 2.094 (10) | --- |
| ESR1 | ligand-dependent nuclear receptor | Promotes | --- | --- | 2.207 (11) | --- |
| Growth hormone | group | Promotes | --- | --- | 2.156 (5) | --- |
| IL15 | cytokine | Inhibits | --- | --- | 2.621 (11) | --- |
| NFkB (complex) | complex | Promotes | --- | --- | 2.103 (20) | --- |
| RHO | G-protein coupled receptor | N.D. | --- | --- | -2.236 (5) | --- |
| TRIM24 | transcription regulator | Promotes | --- | --- | -2.236 (5) | --- |
| CCND1 | other | Promotes | --- | --- | --- | -2.278 (34) |
| CCNK | kinase | Promotes | --- | --- | --- | -2.372 (6) |
| CD3 | complex | Inhibits | --- | --- | --- | 2.443 (75) |
| CREBBP | transcription regulator | Promotes | --- | --- | --- | 2.596 (11) |
| CUL4B | other | Promotes | --- | --- | --- | 2.000 (4) |
| EBI3 | cytokine | Inhibits | --- | --- | --- | 2.213 (5) |
| EIF4G1 | translation regulator | Promotes | --- | --- | --- | -2.000 (4) |
| FOXM1 | transcription regulator | Promotes | --- | --- | --- | -2.358 (15) |
| FOXO1 | transcription regulator | Inhibits | --- | --- | --- | -2.433 (37) |
| GFI1 | transcription regulator | Promotes | --- | --- | --- | -2.236 (6) |
| HDAC2 | transcription regulator | Inhibits | --- | --- | --- | -2.236 (5) |
| HGF | growth factor | Promotes | --- | --- | --- | -2.122 (53) |
| HOXA9 | transcription regulator | Promotes | --- | --- | --- | 2.000 (15) |
| I kappa b kinase | complex | Promotes | --- | --- | --- | 2.224 (5) |
| IL18 | cytokine | Both | --- | --- | --- | 2.182 (10) |
| KLF5 | transcription regulator | Both | --- | --- | --- | 2.196 (5) |
| miR-146a-5p | mature microRNA | Both | --- | --- | --- | 2.135 (8) |
| mir-181 | microRNA | N.D. | --- | --- | --- | -2.000 (4) |
| mir-192 | microRNA | Inhibits | --- | --- | --- | 2.209 (5) |
| miR-483-3p | mature microRNA | Promotes | --- | --- | --- | 2.049 (7) |
| MXI1 | transcription regulator | Inhibits | --- | --- | --- | 2.219 (5) |
| NGF | growth factor | Promotes | --- | --- | --- | 2.245 (10) |
| Pkc(s) | group | Promotes | --- | --- | --- | 2.192 (7) |
| RBL2 | other | Inhibits | --- | --- | --- | 2.777 (17) |
| S100A6 | transporter | Promotes | --- | --- | --- | -2.000 (4) |
| SLC13A1 | transporter | Inhibits | --- | --- | --- | 2.000 (4) |
| SMARCB1 | transcription regulator | Inhibits | --- | --- | --- | 2.383 (16) |
| SPDEF | transcription regulator | Inhibits | --- | --- | --- | -2.177 (5) |
| Tgf beta | group | Both | --- | --- | --- | 2.269 (25) |
| TLR4 | transmembrane receptor | Both | --- | --- | --- | 2.188 (18) |
| TP63 | transcription regulator | Promotes | --- | --- | --- | -2.651 (20) |

**Supplemental Table 3.** Upstream regulators predicted to be activated or inhibited (as indicated by the activation z-score) in the spleen with different age and proton irradiation comparisons (0Gy Adolescent (A), 0Gy Old (O), 0.5Gyx3 Proton Adolescent (A P), 0.5Gyx3 Proton Old (O P)) obtained through the use of Ingenuity Pathway Analysis (IPA) software. Regulation z-score indicates the degree of inhibition (for negative values) or activation (for positive values). The third column denotes the effects the upstream regulators have on tumor progression based on the literature (reference in parenthesis). Upstream regulators which were not determined to have any effects on tumor progression are denoted by N.D.

1. Nakaji, M, Yano, Y, Ninomiya, T, et al. IFN-alpha prevents the growth of pre-neoplastic lesions and inhibits the development of hepatocellular carcinoma in the rat. *Carcinogenesis* 2004;**25**:389-97.

2. Bhattacharya, S, HuangFu, WC, Dong, G, et al. Anti-tumorigenic effects of Type 1 interferon are subdued by integrated stress responses. *Oncogene* 2013;**32**:4214-21.

3. Wood, KJ, Feng, G, Wei, B, et al. Interferon gamma: friend or foe? *Transplantation* 2007;**84**:S4-5.

4. Miller, DM, Thomas, SD, Islam, A, et al. c-Myc and cancer metabolism. *Clin Cancer Res* 2012;**18**:5546-53.

5. Iritani, BM, Eisenman, RN. c-Myc enhances protein synthesis and cell size during B lymphocyte development. *Proc Natl Acad Sci U S A* 1999;**96**:13180-5.

6. Mirzayans, R, Andrais, B, Hansen, G, et al. Role of p16(INK4A) in Replicative Senescence and DNA Damage-Induced Premature Senescence in p53-Deficient Human Cells. *Biochem Res Int* 2012;**2012**:951574.

7. Giacinti, C, Giordano, A. RB and cell cycle progression. *Oncogene* 2006;**25**:5220-7.

8. Wang, B, Lindley, LE, Fernandez-Vega, V, et al. The T box transcription factor TBX2 promotes epithelial-mesenchymal transition and invasion of normal and malignant breast epithelial cells. *PLoS One* 2012;**7**:e41355.

9. Elton, TS, Selemon, H, Elton, SM, et al. Regulation of the MIR155 host gene in physiological and pathological processes. *Gene* 2012.

10. Horowitz, NA, Blevins, EA, Miller, WM, et al. Thrombomodulin is a determinant of metastasis through a mechanism linked to the thrombin binding domain but not the lectin-like domain. *Blood* 2011;**118**:2889-95.

11. Fei, P, Wang, W, Kim, SH, et al. Bnip3L is induced by p53 under hypoxia, and its knockdown promotes tumor growth. *Cancer Cell* 2004;**6**:597-609.

12. Korkaya, H, Kim, GI, Davis, A, et al. Activation of an IL6 inflammatory loop mediates trastuzumab resistance in HER2+ breast cancer by expanding the cancer stem cell population. *Mol Cell* 2012;**47**:570-84.

13. Hayami, S, Yoshimatsu, M, Veerakumarasivam, A, et al. Overexpression of the JmjC histone demethylase KDM5B in human carcinogenesis: involvement in the proliferation of cancer cells through the E2F/RB pathway. *Mol Cancer* 2010;**9**:59.

14. Cellot, S, Hope, KJ, Chagraoui, J, et al. RNAi screen identifies Jarid1b as a major regulator of mouse HSC activity. *Blood* 2013;**122**:1545-55.

15. Hamidi, T, Cano, CE, Grasso, D, et al. NUPR1 works against the metabolic stress-induced autophagy-associated cell death in pancreatic cancer cells. *Autophagy* 2013;**9**:95-7.

16. Chinnam, M, Goodrich, DW. RB1, development, and cancer. *Curr Top Dev Biol* 2011;**94**:129-69.

17. Lara, MF, Santos, M, Ruiz, S, et al. p107 acts as a tumor suppressor in pRb-deficient epidermis. *Mol Carcinog* 2008;**47**:105-13.

18. Mocellin, S, Nitti, D. TNF and cancer: the two sides of the coin. *Front Biosci* 2008;**13**:2774-83.

19. Lehmann, BD, Pietenpol, JA. Targeting mutant p53 in human tumors. *J Clin Oncol* 2012;**30**:3648-50.

20. Herfindal, L, Myhren, L, Gjertsen, BT, et al. Functional p53 is required for rapid restoration of daunorubicin-induced lesions of the spleen. *BMC Cancer* 2013;**13**:341.

21. Sharad, S, Srivastava, A, Ravulapalli, S, et al. Prostate cancer gene expression signature of patients with high body mass index. *Prostate Cancer Prostatic Dis* 2011;**14**:22-9.

22. Barcus, CE, Keely, PJ, Eliceiri, KW, et al. Stiff collagen matrices increase tumorigenic prolactin signaling in breast cancer cells. *J Biol Chem* 2013;**288**:12722-32.

23. Horiguchi, K, Yagi, S, Ono, K, et al. Prolactin gene expression in mouse spleen helper T cells. *J Endocrinol* 2004;**183**:639-46.

24. Naujokat, C, Hoffmann, S. Role and function of the 26S proteasome in proliferation and apoptosis. *Lab Invest* 2002;**82**:965-80.

25. Huang, CY, Fong, YC, Lee, CY, et al. CCL5 increases lung cancer migration via PI3K, Akt and NF-kappaB pathways. *Biochem Pharmacol* 2009;**77**:794-803.

26. Kharas, MG, Okabe, R, Ganis, JJ, et al. Constitutively active AKT depletes hematopoietic stem cells and induces leukemia in mice. *Blood* 2010;**115**:1406-15.

27. Overdevest, JB, Knubel, KH, Duex, JE, et al. CD24 expression is important in male urothelial tumorigenesis and metastasis in mice and is androgen regulated. *Proc Natl Acad Sci U S A* 2012;**109**:E3588-96.

28. Huang, L, Zhao, S, Frasor, JM, et al. An integrated bioinformatics approach identifies elevated cyclin E2 expression and E2F activity as distinct features of tamoxifen resistant breast tumors. *PLoS One* 2011;**6**:e22274.

29. Aoki, I, Higuchi, M, Gotoh, Y. NEDDylation controls the target specificity of E2F1 and apoptosis induction. *Oncogene* 2012.

30. Fuchs, M, Gerber, J, Drapkin, R, et al. The p400 complex is an essential E1A transformation target. *Cell* 2001;**106**:297-307.

31. Dicato, M, Plawny, L. Erythropoietin in cancer patients: pros and cons. *Curr Opin Oncol* 2010;**22**:307-11.

32. Zong, Y, Xin, L, Goldstein, AS, et al. ETS family transcription factors collaborate with alternative signaling pathways to induce carcinoma from adult murine prostate cells. *Proc Natl Acad Sci U S A* 2009;**106**:12465-70.

33. Kohno, M, Pouyssegur, J. Targeting the ERK signaling pathway in cancer therapy. *Ann Med* 2006;**38**:200-11.

34. Guihard, S, Clay, D, Cocault, L, et al. The MAPK ERK1 is a negative regulator of the adult steady-state splenic erythropoiesis. *Blood* 2010;**115**:3686-94.

35. Deblois, G, Chahrour, G, Perry, MC, et al. Transcriptional control of the ERBB2 amplicon by ERRalpha and PGC-1beta promotes mammary gland tumorigenesis. *Cancer Res* 2010;**70**:10277-87.

36. Jiang, N, Zhou, LQ, Zhang, XY. Downregulation of the nucleosome-binding protein 1 (NSBP1) gene can inhibit the in vitro and in vivo proliferation of prostate cancer cells. *Asian J Androl* 2010;**12**:709-17.

37. Chariot, A. The NF-kappaB-independent functions of IKK subunits in immunity and cancer. *Trends Cell Biol* 2009;**19**:404-13.

38. Apte, RN, Dotan, S, Elkabets, M, et al. The involvement of IL-1 in tumorigenesis, tumor invasiveness, metastasis and tumor-host interactions. *Cancer Metastasis Rev* 2006;**25**:387-408.

39. Fernandez-Zapico, ME, Lomberk, GA, Tsuji, S, et al. A functional family-wide screening of SP/KLF proteins identifies a subset of suppressors of KRAS-mediated cell growth. *Biochem J* 2011;**435**:529-37.

40. Niture, SK, Jaiswal, AK. Nrf2 protein up-regulates antiapoptotic protein Bcl-2 and prevents cellular apoptosis. *J Biol Chem* 2012;**287**:9873-86.

41. Charafe-Jauffret, E, Ginestier, C, Iovino, F, et al. Breast cancer cell lines contain functional cancer stem cells with metastatic capacity and a distinct molecular signature. *Cancer Res* 2009;**69**:1302-13.

42. Chow, MT, Sceneay, J, Paget, C, et al. NLRP3 suppresses NK cell-mediated responses to carcinogen-induced tumors and metastases. *Cancer Res* 2012;**72**:5721-32.

43. Licciulli, S, Avila, JL, Hanlon, L, et al. Notch1 is required for Kras-induced lung adenocarcinoma and controls tumor cell survival via p53. *Cancer Res* 2013;**73**:5974-84.

44. Radtke, F, Fasnacht, N, Macdonald, HR. Notch signaling in the immune system. *Immunity* 2010;**32**:14-27.

45. Sollazzo, V, Galasso, M, Volinia, S, et al. Prion proteins (PRNP and PRND) are over-expressed in osteosarcoma. *J Orthop Res* 2012;**30**:1004-12.

46. Knobbe, CB, Lapin, V, Suzuki, A, et al. The roles of PTEN in development, physiology and tumorigenesis in mouse models: a tissue-by-tissue survey. *Oncogene* 2008;**27**:5398-415.

47. Romero, OA, Setien, F, John, S, et al. The tumour suppressor and chromatin-remodelling factor BRG1 antagonizes Myc activity and promotes cell differentiation in human cancer. *EMBO Mol Med* 2012;**4**:603-16.

48. Wu, J, Patmore, DM, Jousma, E, et al. EGFR-STAT3 signaling promotes formation of malignant peripheral nerve sheath tumors. *Oncogene* 2013.

49. Hossain, DM, Dos Santos, C, Zhang, Q, et al. Leukemia cell-targeted STAT3 silencing and TLR9 triggering generate systemic antitumor immunity. *Blood* 2014;**123**:15-25.

50. Slyper, M, Shahar, A, Bar-Ziv, A, et al. Control of breast cancer growth and initiation by the stem cell-associated transcription factor TCF3. *Cancer Res* 2012;**72**:5613-24.

51. van der Veer, A, van der Velden, VH, Willemse, ME, et al. Interference with pre-B-cell receptor signaling offers a therapeutic option for TCF3-rearranged childhood acute lymphoblastic leukemia. *Blood Cancer J* 2014;**4**:e181.

52. Johnson, LA, Morgan, RA, Dudley, ME, et al. Gene therapy with human and mouse T-cell receptors mediates cancer regression and targets normal tissues expressing cognate antigen. *Blood* 2009;**114**:535-46.

53. Baek, JY, Morris, SM, Campbell, J, et al. TGF-beta inactivation and TGF-alpha overexpression cooperate in an in vivo mouse model to induce hepatocellular carcinoma that recapitulates molecular features of human liver cancer. *Int J Cancer* 2010;**127**:1060-71.

54. Nguyen, DH, Martinez-Ruiz, H, Barcellos-Hoff, MH. Consequences of epithelial or stromal TGFbeta1 depletion in the mammary gland. *J Mammary Gland Biol Neoplasia* 2011;**16**:147-55.

55. Rakoff-Nahoum, S, Medzhitov, R. Toll-like receptors and cancer. *Nat Rev Cancer* 2009;**9**:57-63.

56. Salaun, B, Coste, I, Rissoan, MC, et al. TLR3 can directly trigger apoptosis in human cancer cells. *J Immunol* 2006;**176**:4894-901.

57. El Andaloussi, A, Sonabend, AM, Han, Y, et al. Stimulation of TLR9 with CpG ODN enhances apoptosis of glioma and prolongs the survival of mice with experimental brain tumors. *Glia* 2006;**54**:526-35.

58. Onji, M, Kanno, A, Saitoh, S, et al. An essential role for the N-terminal fragment of Toll-like receptor 9 in DNA sensing. *Nat Commun* 2013;**4**:1949.

59. Bigarella, CL, Borges, L, Costa, FF, et al. ARHGAP21 modulates FAK activity and impairs glioblastoma cell migration. *Biochim Biophys Acta* 2009;**1793**:806-16.

60. Naor, D, Nedvetzki, S, Golan, I, et al. CD44 in cancer. *Crit Rev Clin Lab Sci* 2002;**39**:527-79.

61. Brideau, G, Makinen, MJ, Elamaa, H, et al. Endostatin overexpression inhibits lymphangiogenesis and lymph node metastasis in mice. *Cancer Res* 2007;**67**:11528-35.

62. Roberti, MP, Arriaga, JM, Bianchini, M, et al. Protein expression changes during human triple negative breast cancer cell line progression to lymph node metastasis in a xenografted model in nude mice. *Cancer Biol Ther* 2012;**13**:1123-40.

63. Hattermann, K, Mentlein, R. An infernal trio: the chemokine CXCL12 and its receptors CXCR4 and CXCR7 in tumor biology. *Ann Anat* 2013;**195**:103-10.

64. Lam, DC, Girard, L, Ramirez, R, et al. Expression of nicotinic acetylcholine receptor subunit genes in non-small-cell lung cancer reveals differences between smokers and nonsmokers. *Cancer Res* 2007;**67**:4638-47.

65. Mori, S, Tran, V, Nishikawa, K, et al. A dominant-negative FGF1 mutant (the R50E mutant) suppresses tumorigenesis and angiogenesis. *PLoS One* 2013;**8**:e57927.

66. Hellebrekers, DM, Lentjes, MH, van den Bosch, SM, et al. GATA4 and GATA5 are potential tumor suppressors and biomarkers in colorectal cancer. *Clin Cancer Res* 2009;**15**:3990-7.

67. Onda, M, Akaishi, J, Asaka, S, et al. Decreased expression of haemoglobin beta (HBB) gene in anaplastic thyroid cancer and recovery of its expression inhibits cell growth. *Br J Cancer* 2005;**92**:2216-24.

68. Chiavarina, B, Whitaker-Menezes, D, Migneco, G, et al. HIF1-alpha functions as a tumor promoter in cancer associated fibroblasts, and as a tumor suppressor in breast cancer cells: Autophagy drives compartment-specific oncogenesis. *Cell Cycle* 2010;**9**:3534-51.

69. Corzo, CA, Condamine, T, Lu, L, et al. HIF-1alpha regulates function and differentiation of myeloid-derived suppressor cells in the tumor microenvironment. *J Exp Med* 2010;**207**:2439-53.

70. Wagner, EF, Nebreda, AR. Signal integration by JNK and p38 MAPK pathways in cancer development. *Nat Rev Cancer* 2009;**9**:537-49.

71. Khaidakov, M, Mehta, JL. Oxidized LDL triggers pro-oncogenic signaling in human breast mammary epithelial cells partly via stimulation of MiR-21. *PLoS One* 2012;**7**:e46973.

72. Qiu, MH, Qian, YM, Zhao, XL, et al. Expression and prognostic significance of MAP4K4 in lung adenocarcinoma. *Pathol Res Pract* 2012;**208**:541-8.

73. Hatakeyama, M. Helicobacter pylori and gastric carcinogenesis. *J Gastroenterol* 2009;**44**:239-48.

74. Lindeman, GJ, Harris, AW, Bath, ML, et al. Overexpressed max is not oncogenic and attenuates myc-induced lymphoproliferation and lymphomagenesis in transgenic mice. *Oncogene* 1995;**10**:1013-7.

75. Casey, SC, Blumberg, B. The steroid and xenobiotic receptor negatively regulates B-1 cell development in the fetal liver. *Mol Endocrinol* 2012;**26**:916-25.

76. McPhillips, F, Mullen, P, MacLeod, KG, et al. Raf-1 is the predominant Raf isoform that mediates growth factor-stimulated growth in ovarian cancer cells. *Carcinogenesis* 2006;**27**:729-39.

77. Meng, Q, Zheng, M, Liu, H, et al. SASH1 regulates proliferation, apoptosis, and invasion of osteosarcoma cell. *Mol Cell Biochem* 2013;**373**:201-10.

78. Coupland, LA, Chong, BH, Parish, CR. Platelets and P-selectin control tumor cell metastasis in an organ-specific manner and independently of NK cells. *Cancer Res* 2012;**72**:4662-71.

79. Yamaoka, T, Fujimoto, M, Ogawa, F, et al. The roles of P- and E-selectins and P-selectin glycoprotein ligand-1 in primary and metastatic mouse melanomas. *J Dermatol Sci* 2011;**64**:99-107.

80. Cook, WD, McCaw, BJ, Herring, C, et al. PU.1 is a suppressor of myeloid leukemia, inactivated in mice by gene deletion and mutation of its DNA binding domain. *Blood* 2004;**104**:3437-44.

81. Yu, J, Ma, X, Cheung, KF, et al. Epigenetic inactivation of T-box transcription factor 5, a novel tumor suppressor gene, is associated with colon cancer. *Oncogene* 2010;**29**:6464-74.

82. Rufini, A, Agostini, M, Grespi, F, et al. p73 in Cancer. *Genes Cancer* 2011;**2**:491-502.

83. Shin, M, Mizokami, A, Kim, J, et al. Exogenous SPARC suppresses proliferation and migration of prostate cancer by interacting with integrin beta1. *Prostate* 2013;**73**:1159-70.

84. Rempel, SA, Hawley, RC, Gutierrez, JA, et al. Splenic and immune alterations of the Sparc-null mouse accompany a lack of immune response. *Genes Immun* 2007;**8**:262-74.

85. Hix, LM, Karavitis, J, Khan, MW, et al. Tumor STAT1 Transcription Factor Activity Enhances Breast Tumor Growth and Immune Suppression Mediated by Myeloid-derived Suppressor Cells. *J Biol Chem* 2013;**288**:11676-88.

86. Mitsuhashi, M, Peel, D, Ziogas, A, et al. Enhanced Expression of Radiation-induced Leukocyte CDKN1A mRNA in Multiple Primary Breast Cancer Patients: Potential New Marker of Cancer Susceptibility. *Biomark Insights* 2009;**4**:201-9.

87. Uemura, Y, Kobayashi, M, Nakata, H, et al. Effects of GM-CSF and M-CSF on tumor progression of lung cancer: roles of MEK1/ERK and AKT/PKB pathways. *Int J Mol Med* 2006;**18**:365-73.

88. Scharenberg, MA, Chiquet-Ehrismann, R, Asparuhova, MB. Megakaryoblastic leukemia protein-1 (MKL1): Increasing evidence for an involvement in cancer progression and metastasis. *Int J Biochem Cell Biol* 2010;**42**:1911-4.

89. Calao, M, Sekyere, EO, Cui, HJ, et al. Direct effects of Bmi1 on p53 protein stability inactivates oncoprotein stress responses in embryonal cancer precursor cells at tumor initiation. *Oncogene* 2013;**32**:3616-26.

90. Sutherland, M, Gill, JH, Loadman, PM, et al. Antitumor Activity of a Duocarmycin Analogue Rationalised to Be Metabolically Activated by Cytochrome P450 1a1 in Human Transitional Cell Carcinoma of the Bladder. *Mol Cancer Ther* 2012.

91. Franco, CA, Blanc, J, Parlakian, A, et al. SRF selectively controls tip cell invasive behavior in angiogenesis. *Development* 2013;**140**:2321-33.

92. Rodrigues-Ferreira, S, Abdelkarim, M, Dillenburg-Pilla, P, et al. Angiotensin II facilitates breast cancer cell migration and metastasis. *PLoS One* 2012;**7**:e35667.

93. Hansson, ML, Behmer, S, Ceder, R, et al. MAML1 acts cooperatively with EGR1 to activate EGR1-regulated promoters: implications for nephrogenesis and the development of renal cancer. *PLoS One* 2012;**7**:e46001.

94. Simson, L, Ellyard, JI, Dent, LA, et al. Regulation of carcinogenesis by IL-5 and CCL11: a potential role for eosinophils in tumor immune surveillance. *J Immunol* 2007;**178**:4222-9.

95. Bouker, KB, Skaar, TC, Riggins, RB, et al. Interferon regulatory factor-1 (IRF-1) exhibits tumor suppressor activities in breast cancer associated with caspase activation and induction of apoptosis. *Carcinogenesis* 2005;**26**:1527-35.

96. Owens, BM, Moore, JW, Kaye, PM. IRF7 regulates TLR2-mediated activation of splenic CD11c(hi) dendritic cells. *PLoS One* 2012;**7**:e41050.

97. Wang, XM, Li, J, Yan, MX, et al. Integrative analyses identify osteopontin, LAMB3 and ITGB1 as critical pro-metastatic genes for lung cancer. *PLoS One* 2013;**8**:e55714.

98. Dabizzi, S, Noci, I, Borri, P, et al. Luteinizing hormone increases human endometrial cancer cells invasiveness through activation of protein kinase A. *Cancer Res* 2003;**63**:4281-6.

99. Chen, P, O'Neal, JF, Ebelt, ND, et al. Jnk2 effects on tumor development, genetic instability and replicative stress in an oncogene-driven mouse mammary tumor model. *PLoS One* 2010;**5**:e10443.

100. Salama, RH, Muramatsu, H, Zou, P, et al. Midkine, a heparin-binding growth factor, produced by the host enhances metastasis of Lewis lung carcinoma cells. *Cancer Lett* 2006;**233**:16-20.

101. Nguyen, MH, Koinuma, J, Ueda, K, et al. Phosphorylation and activation of cell division cycle associated 5 by mitogen-activated protein kinase play a crucial role in human lung carcinogenesis. *Cancer Res* 2010;**70**:5337-47.

102. Yu, W, Lin, Z, Pastor, DM, et al. Genes regulated by Nkx2-3 in sporadic and inflammatory bowel disease-associated colorectal cancer cell lines. *Dig Dis Sci* 2010;**55**:3171-80.

103. Czompoly, T, Labadi, A, Kellermayer, Z, et al. Transcription factor Nkx2-3 controls the vascular identity and lymphocyte homing in the spleen. *J Immunol* 2011;**186**:6981-9.

104. Cheng, J, Ye, H, Liu, Z, et al. Platelet-derived growth factor-BB accelerates prostate cancer growth by promoting the proliferation of mesenchymal stem cells. *J Cell Biochem* 2013.

105. Xue, Y, Lim, S, Yang, Y, et al. PDGF-BB modulates hematopoiesis and tumor angiogenesis by inducing erythropoietin production in stromal cells. *Nat Med* 2012;**18**:100-10.

106. Takahashi, K, Takeda, K, Saiki, I, et al. Functional roles of tumor necrosis factor-related apoptosis-inducing ligand-DR5 interaction in B16F10 cells by activating the nuclear factor-kappaB pathway to induce metastatic potential. *Cancer Sci* 2013;**104**:558-62.

107. Wu, J, Li, J, Salcedo, R, et al. The proinflammatory myeloid cell receptor TREM-1 controls Kupffer cell activation and development of hepatocellular carcinoma. *Cancer Res* 2012;**72**:3977-86.

108. Natividad, KD, Junankar, SR, Mohd Redzwan, N, et al. Interleukin-27 signaling promotes immunity against endogenously arising murine tumors. *PLoS One* 2013;**8**:e57469.

109. Romieu-Mourez, R, Solis, M, Nardin, A, et al. Distinct roles for IFN regulatory factor (IRF)-3 and IRF-7 in the activation of antitumor properties of human macrophages. *Cancer Res* 2006;**66**:10576-85.

110. Sun, X, Qin, S, Fan, C, et al. Let-7: A regulator of the ERalpha signaling pathway in human breast tumors and breast cancer stem cells. *Oncol Rep* 2013.

111. Staehli, F, Ludigs, K, Heinz, LX, et al. NLRC5 deficiency selectively impairs MHC class I- dependent lymphocyte killing by cytotoxic T cells. *J Immunol* 2012;**188**:3820-8.

112. Miyoshi, N, Ishii, H, Mimori, K, et al. TGM2 is a novel marker for prognosis and therapeutic target in colorectal cancer. *Ann Surg Oncol* 2010;**17**:967-72.

113. Peterson, JE, Zurakowski, D, Italiano, JE, Jr., et al. VEGF, PF4 and PDGF are elevated in platelets of colorectal cancer patients. *Angiogenesis* 2012;**15**:265-73.

114. Duan, L, Ye, L, Zhao, G, et al. Serum spleen tyrosine kinase and vascular endothelial growth factor-C levels predict lymph node metastasis of oesophageal squamous cell carcinoma. *Eur J Cardiothorac Surg* 2013;**43**:e58-63.

115. Chakravarty, D, Nair, SS, Santhamma, B, et al. Extranuclear functions of ER impact invasive migration and metastasis by breast cancer cells. *Cancer Res* 2010;**70**:4092-101.

116. Holly, JM, Gunnell, DJ, Davey Smith, G. Growth hormone, IGF-I and cancer. Less intervention to avoid cancer? More intervention to prevent cancer? *J Endocrinol* 1999;**162**:321-30.

117. Steel, JC, Waldmann, TA, Morris, JC. Interleukin-15 biology and its therapeutic implications in cancer. *Trends Pharmacol Sci* 2012;**33**:35-41.

118. Alberti, C, Pinciroli, P, Valeri, B, et al. Ligand-dependent EGFR activation induces the co-expression of IL-6 and PAI-1 via the NFkB pathway in advanced-stage epithelial ovarian cancer. *Oncogene* 2012;**31**:4139-49.

119. Li, H, Sun, L, Tang, Z, et al. Overexpression of TRIM24 correlates with tumor progression in non-small cell lung cancer. *PLoS One* 2012;**7**:e37657.

120. Cao, L, Li, C, Shen, S, et al. OCT4 increases BIRC5 and CCND1 expression and promotes cancer progression in hepatocellular carcinoma. *BMC Cancer* 2013;**13**:82.

121. Marsaud, V, Tchakarska, G, Andrieux, G, et al. Cyclin K and cyclin D1b are oncogenic in myeloma cells. *Mol Cancer* 2010;**9**:103.

122. Nakajima, F, Khanna, A, Xu, G, et al. Immunotherapy with anti-CD3 monoclonal antibodies and recombinant interleukin 2: stimulation of molecular programs of cytotoxic killer cells and induction of tumor regression. *Proc Natl Acad Sci U S A* 1994;**91**:7889-93.

123. Inthal, A, Zeitlhofer, P, Zeginigg, M, et al. CREBBP HAT domain mutations prevail in relapse cases of high hyperdiploid childhood acute lymphoblastic leukemia. *Leukemia* 2012;**26**:1797-803.

124. Jiang, T, Tang, HM, Wu, ZH, et al. Cullin 4B is a novel prognostic marker that correlates with colon cancer progression and pathogenesis. *Med Oncol* 2013;**30**:534.

125. Liu, Z, Liu, JQ, Talebian, F, et al. IL-27 enhances the survival of tumor antigen-specific CD8+ T cells and programs them into IL-10-producing, memory precursor-like effector cells. *Eur J Immunol* 2013;**43**:468-79.

126. Tu, L, Liu, Z, He, X, et al. Over-expression of eukaryotic translation initiation factor 4 gamma 1 correlates with tumor progression and poor prognosis in nasopharyngeal carcinoma. *Mol Cancer* 2010;**9**:78.

127. Xu, N, Jia, D, Chen, W, et al. FoxM1 Is Associated with Poor Prognosis of Non-Small Cell Lung Cancer Patients through Promoting Tumor Metastasis. *PLoS One* 2013;**8**:e59412.

128. Motiwala, T, Kutay, H, Zanesi, N, et al. PTPROt-mediated regulation of p53/Foxm1 suppresses leukemic phenotype in a CLL mouse model. *Leukemia* 2014.

129. Zhang, H, Pan, Y, Zheng, L, et al. FOXO1 inhibits Runx2 transcriptional activity and prostate cancer cell migration and invasion. *Cancer Res* 2011;**71**:3257-67.

130. Khandanpour, C, Phelan, JD, Vassen, L, et al. Growth factor independence 1 antagonizes a p53-induced DNA damage response pathway in lymphoblastic leukemia. *Cancer Cell* 2013;**23**:200-14.

131. Heideman, MR, Wilting, RH, Yanover, E, et al. Dosage-dependent tumor suppression by histone deacetylases 1 and 2 through regulation of c-Myc collaborating genes and p53 function. *Blood* 2013;**121**:2038-50.

132. Cecchi, F, Rabe, DC, Bottaro, DP. Targeting the HGF/Met signaling pathway in cancer therapy. *Expert Opin Ther Targets* 2012;**16**:553-72.

133. Beachy, SH, Onozawa, M, Silverman, D, et al. Isolated Hoxa9 overexpression predisposes to the development of lymphoid but not myeloid leukemia. *Exp Hematol* 2013;**41**:518-29 e5.

134. Zhou, AY, Shen, RR, Kim, E, et al. IKKepsilon-mediated tumorigenesis requires K63-linked polyubiquitination by a cIAP1/cIAP2/TRAF2 E3 ubiquitin ligase complex. *Cell Rep* 2013;**3**:724-33.

135. Kuppala, MB, Syed, SB, Bandaru, S, et al. Immunotherapeutic approach for better management of cancer--role of IL-18. *Asian Pac J Cancer Prev* 2012;**13**:5353-61.

136. Bialkowska, AB, Crisp, M, Bannister, T, et al. Identification of small-molecule inhibitors of the colorectal cancer oncogene Kruppel-like factor 5 expression by ultrahigh-throughput screening. *Mol Cancer Ther* 2011;**10**:2043-51.

137. Taniguchi Ishikawa, E, Chang, KH, Nayak, R, et al. Klf5 controls bone marrow homing of stem cells and progenitors through Rab5-mediated beta1/beta2-integrin trafficking. *Nat Commun* 2013;**4**:1660.

138. Mei, J, Bachoo, R, Zhang, CL. MicroRNA-146a inhibits glioma development by targeting Notch1. *Mol Cell Biol* 2011;**31**:3584-92.

139. Feng, S, Cong, S, Zhang, X, et al. MicroRNA-192 targeting retinoblastoma 1 inhibits cell proliferation and induces cell apoptosis in lung cancer cells. *Nucleic Acids Res* 2011;**39**:6669-78.

140. Veronese, A, Lupini, L, Consiglio, J, et al. Oncogenic role of miR-483-3p at the IGF2/483 locus. *Cancer Res* 2010;**70**:3140-9.

141. Schreiber-Agus, N, Meng, Y, Hoang, T, et al. Role of Mxi1 in ageing organ systems and the regulation of normal and neoplastic growth. *Nature* 1998;**393**:483-7.

142. Adriaenssens, E, Vanhecke, E, Saule, P, et al. Nerve growth factor is a potential therapeutic target in breast cancer. *Cancer Res* 2008;**68**:346-51.

143. Hsu, FM, Zhang, S, Chen, BP. Role of DNA-dependent protein kinase catalytic subunit in cancer development and treatment. *Transl Cancer Res* 2012;**1**:22-34.

144. Indovina, P, Marcelli, E, Casini, N, et al. Emerging roles of RB family: new defense mechanisms against tumor progression. *J Cell Physiol* 2013;**228**:525-35.

145. Ning, X, Sun, S, Zhang, K, et al. S100A6 protein negatively regulates CacyBP/SIP-mediated inhibition of gastric cancer cell proliferation and tumorigenesis. *PLoS One* 2012;**7**:e30185.

146. Dawson, PA, Choyce, A, Chuang, C, et al. Enhanced tumor growth in the NaS1 sulfate transporter null mouse. *Cancer Sci* 2010;**101**:369-73.

147. Modena, P, Lualdi, E, Facchinetti, F, et al. SMARCB1/INI1 tumor suppressor gene is frequently inactivated in epithelioid sarcomas. *Cancer Res* 2005;**65**:4012-9.

148. Noah, TK, Lo, YH, Price, A, et al. SPDEF functions as a colorectal tumor suppressor by inhibiting beta-catenin activity. *Gastroenterology* 2013;**144**:1012-23 e6.

149. Oblak, A, Jerala, R. Toll-like receptor 4 activation in cancer progression and therapy. *Clin Dev Immunol* 2011;**2011**:609579.

150. Graziano, V, De Laurenzi, V. Role of p63 in cancer development. *Biochim Biophys Acta* 2011;**1816**:57-66.
