# Supplementary material for: Proton irradiation impacts age-driven modulations of cancer progression influenced by immune system transcriptome modifications from splenic tissue
Source: J Radiat Res. 2015 Aug 7;56(5):792–803. doi: 10.1093/jrr/rrv043 (PMC4577010; doi:10.1093/jrr/rrv043)
Supplement: Supplementary Data [file supp_rrv043_rrv043supp_table4.doc]

| **Gene Name** | **Group** | **Effects on Tumor (Ref.)** | **Log_2_ Fold Change** | | | |
| --- | --- | --- | --- | --- | --- | --- |
|  |  |  | **A P vs A** | **O P vs O** | **O vs A** | **O P vs A P** |
| CD74 | A P vs A & O P vs O & O P vs A P & O vs A | Promotes | -0.4463 | 0.2688 | 0.3579 | 1.0730 |
| MCM7 | A P vs A & O P vs O & O P vs A P | Promotes | 0.3849 | -0.3348 | -0.1696 | -0.8892 |
| NDE1 | A P vs A | Promotes | 0.5554 | 0.1847 | -0.1283 | -0.4990 |
| RFC1 | A P vs A | Promotes | 0.3231 | -0.0388 | -0.1453 | -0.5071 |
| RFC3 | A P vs A | Promotes | 0.5264 | 0.0020 | -0.0673 | -0.5917 |
| SIRT2 | A P vs A | Inhibits | 0.3334 | 0.4148 | 0.0157 | 0.0971 |
| ASNS | A P vs A & O P vs A P | Promotes | 0.7715 | -0.5730 | -0.3854 | -1.7299 |
| ATP5B | A P vs A & O P vs A P | Inhibits | 0.3196 | 0.0016 | -0.1234 | -0.4414 |
| BLM | A P vs A & O P vs A P | Inhibits | 0.6053 | -0.2980 | -0.2534 | -1.1568 |
| BUB1B | A P vs A & O P vs A P | Inhibits | 0.4112 | -0.1925 | -0.2652 | -0.8689 |
| CAD | A P vs A & O P vs A P | Promotes | 0.5340 | 0.2156 | -0.1079 | -0.4263 |
| CDC20 | A P vs A & O P vs A P | Promotes | 0.5534 | -0.0127 | -0.3404 | -0.9065 |
| CDC25A | A P vs A & O P vs A P | Promotes | 0.4375 | -0.0518 | -0.2722 | -0.7615 |
| CDCA5 | A P vs A & O P vs A P | Promotes | 0.4326 | -0.2534 | -0.2201 | -0.9061 |
| CDK2 | A P vs A & O P vs A P | Promotes | 0.7938 | 0.0417 | -0.1329 | -0.8850 |
| CIITA | A P vs A & O P vs A P | Inhibits | -0.6351 | 0.5626 | 0.3792 | 1.5769 |
| KARS | A P vs A & O P vs A P | Inhibits | 0.4949 | 0.0667 | -0.0309 | -0.4592 |
| KIF11 | A P vs A & O P vs A P | Promotes | 0.9483 | 0.3982 | -0.2000 | -0.7500 |
| KNTC1 | A P vs A & O P vs A P | Inhibits | 0.8420 | -0.1233 | -0.2156 | -1.1808 |
| LSM3 | A P vs A & O P vs A P | Promotes | 0.2904 | -0.1758 | -0.1448 | -0.6110 |
| MAD2L1 | A P vs A & O P vs A P | Promotes | 0.5843 | -0.1273 | -0.1996 | -0.9113 |
| MCM2 | A P vs A & O P vs A P | Promotes | 0.4558 | -0.1329 | -0.3201 | -0.9088 |
| MCM3 | A P vs A & O P vs A P | Promotes | 0.9096 | 0.0324 | -0.2014 | -1.0786 |
| MYH10 | A P vs A & O P vs A P | N.D. | 0.9863 | 0.0065 | -0.1648 | -1.1447 |
| MYH9 | A P vs A & O P vs A P | Promotes | -0.5490 | 0.0956 | 0.1057 | 0.7502 |
| MYO9B | A P vs A & O P vs A P | Promotes | -0.7286 | 0.0802 | 0.2097 | 1.0185 |
| NDC80 | A P vs A & O P vs A P | Promotes | 0.3918 | -0.0624 | -0.1245 | -0.5787 |
| NUP62 | A P vs A & O P vs A P | N.D. | -0.3291 | -0.7935 | -0.0731 | -0.5374 |
| PSMB10 | A P vs A & O P vs A P | Inhibits | -0.4475 | 0.1646 | 0.3206 | 0.9327 |
| RAD17 | A P vs A & O P vs A P | Inhibits | 0.3784 | 0.0660 | -0.0270 | -0.3395 |
| RUVBL1 | A P vs A & O P vs A P | Promotes | 0.3134 | -0.0694 | -0.1446 | -0.5273 |
| RUVBL2 | A P vs A & O P vs A P | Promotes | 0.4065 | -0.1141 | -0.0986 | -0.6192 |
| SNRPD3 | A P vs A & O P vs A P | Inhibits | 0.9013 | 0.4036 | -0.1942 | -0.6919 |
| SNRPF | A P vs A & O P vs A P | Promotes | 0.5487 | 0.2579 | -0.1214 | -0.4122 |
| TOP1 | A P vs A & O P vs A P | Promotes | 0.7991 | -0.0250 | -0.0791 | -0.9032 |
| TOP2A | A P vs A & O P vs A P | Promotes | 0.3025 | -0.7523 | -0.1749 | -1.2298 |
| XRCC2 | A P vs A & O P vs A P | Inhibits | 0.3247 | -0.0956 | -0.0495 | -0.4698 |
| XRCC6 | A P vs A & O P vs A P | Both | 0.4291 | -0.2120 | -0.1300 | -0.7711 |
| ZW10 | A P vs A & O P vs A P | Promotes | 0.4855 | 0.1074 | -0.2046 | -0.5827 |
| ZWINT | A P vs A & O P vs A P | Promotes | 0.2666 | -0.3600 | -0.0869 | -0.7135 |
| MYH11 | O P vs O & O P vs A P | Promotes | 0.0225 | 0.5157 | 0.0169 | 0.5101 |
| NCL | O P vs O & O P vs A P | Promotes | -0.4530 | -0.7155 | -0.1862 | -0.4487 |
| RAD51 | O P vs O & O P vs A P | Promotes | 0.1941 | -0.2711 | -0.1868 | -0.6521 |
| HSPD1 | O P vs A P & O vs A | Promotes | -0.1412 | -0.4963 | -0.3755 | -0.7307 |
| CD274 | O vs A | Inhibits | -0.4238 | 0.2384 | 0.5455 | 1.2077 |
| IL27RA | O vs A | Inhibits | -0.7667 | 0.1159 | 0.3408 | 1.2234 |
| LAG3 | O vs A | Promotes | -0.4040 | -0.2760 | 0.6555 | 0.7835 |
| TNFRSF4 | O vs A | Inhibits | -0.7691 | 0.1237 | 0.3886 | 1.2814 |
| AURKA | O P vs A P | Promotes | -0.3883 | -0.7310 | -0.4129 | -0.7556 |
| CDC7 | O P vs A P | Promotes | 0.1337 | -0.2567 | -0.3145 | -0.7049 |
| CHEK1 | O P vs A P | Promotes | 0.1833 | -0.0439 | -0.0664 | -0.2936 |
| HSP90B1 | O P vs A P | Promotes | -1.0080 | -0.5056 | 0.1325 | 0.6349 |
| KIF2C | O P vs A P | Promotes | 0.3867 | -0.1434 | -0.2898 | -0.8199 |
| LONP1 | O P vs A P | Promotes | 0.5092 | 0.3237 | -0.1656 | -0.3512 |
| NLRC4 | O P vs A P | Promotes | -0.1521 | 0.1853 | 0.1760 | 0.5135 |
| NME4 | O P vs A P | Inhibits | 0.6123 | -0.1168 | -0.0843 | -0.8134 |
| NUSAP1 | O P vs A P | Promotes | 0.2517 | -0.0032 | -0.0396 | -0.2944 |
| PRPS1 | O P vs A P | N.D. | -0.1732 | -0.6509 | -0.1873 | -0.6651 |
| RAD51C | O P vs A P | Inhibits | 0.2187 | -0.3009 | -0.1568 | -0.6764 |
| RECQL4 | O P vs A P | Promotes | 0.1939 | -0.0999 | -0.1605 | -0.4544 |
| STK11 | O P vs A P | Inhibits | -0.2729 | -0.6117 | -0.3079 | -0.6467 |

**Supplemental Table 4.** Key genes involved in age-dependent spleen changes with proton irradiation. These key genes were determined by finding the common genes in the spleen with different age and proton irradiation comparisons (0Gy Adolescent (A), 0Gy Old (O), 0.5Gyx3 Proton Adolescent (A P), 0.5Gyx3 Proton Old (O P)) that are in common between the significant upstream regulators, the biofunction analysis, Gene Set Enrichment Analysis (GSEA) for GO gene sets with a FDR < 0.05, and genes involved in the top 10 functional annotation clusters in DAVID for each group. The second column denotes the effects these genes have on tumor progression based on the literature. Genes with no reported effects on tumors were identified as Not Determined (ND).

1. Choi, JW, Kim, Y, Lee, JH, et al. CD74 expression is increased in high-grade, invasive urothelial carcinoma of the bladder. *Int J Urol* 2013;**20**:251-5.

2. Fristrup, N, Birkenkamp-Demtroder, K, Reinert, T, et al. Multicenter validation of cyclin D1, MCM7, TRIM29, and UBE2C as prognostic protein markers in non-muscle-invasive bladder cancer. *Am J Pathol* 2013;**182**:339-49.

3. Yan, X, Li, F, Liang, Y, et al. Human Nudel and NudE as regulators of cytoplasmic dynein in poleward protein transport along the mitotic spindle. *Mol Cell Biol* 2003;**23**:1239-50.

4. Galbiatti, AL, Ruiz, MT, Maniglia, JV, et al. Head and neck cancer: genetic polymorphisms and folate metabolism. *Braz J Otorhinolaryngol* 2012;**78**:132-9.

5. Lockwood, WW, Thu, KL, Lin, L, et al. Integrative genomics identified RFC3 as an amplified candidate oncogene in esophageal adenocarcinoma. *Clin Cancer Res* 2012;**18**:1936-46.

6. Kim, HS, Vassilopoulos, A, Wang, RH, et al. SIRT2 maintains genome integrity and suppresses tumorigenesis through regulating APC/C activity. *Cancer Cell* 2011;**20**:487-99.

7. Balasubramanian, MN, Butterworth, EA, Kilberg, MS. Asparagine synthetase: regulation by cell stress and involvement in tumor biology. *Am J Physiol Endocrinol Metab* 2013;**304**:E789-99.

8. Sanchez-Arago, M, Chamorro, M, Cuezva, JM. Selection of cancer cells with repressed mitochondria triggers colon cancer progression. *Carcinogenesis* 2010;**31**:567-76.

9. Davari, P, Hebert, JL, Albertson, DG, et al. Loss of Blm enhances basal cell carcinoma and rhabdomyosarcoma tumorigenesis in Ptch1+/- mice. *Carcinogenesis* 2010;**31**:968-73.

10. Baker, DJ, Dawlaty, MM, Wijshake, T, et al. Increased expression of BubR1 protects against aneuploidy and cancer and extends healthy lifespan. *Nat Cell Biol* 2013;**15**:96-102.

11. Otto, E, McCord, S, Tlsty, TD. Increased incidence of CAD gene amplification in tumorigenic rat lines as an indicator of genomic instability of neoplastic cells. *J Biol Chem* 1989;**264**:3390-6.

12. Kidokoro, T, Tanikawa, C, Furukawa, Y, et al. CDC20, a potential cancer therapeutic target, is negatively regulated by p53. *Oncogene* 2008;**27**:1562-71.

13. Shen, T, Huang, S. The role of Cdc25A in the regulation of cell proliferation and apoptosis. *Anticancer Agents Med Chem* 2012;**12**:631-9.

14. Nguyen, MH, Koinuma, J, Ueda, K, et al. Phosphorylation and activation of cell division cycle associated 5 by mitogen-activated protein kinase play a crucial role in human lung carcinogenesis. *Cancer Res* 2010;**70**:5337-47.

15. Tetsu, O, McCormick, F. Proliferation of cancer cells despite CDK2 inhibition. *Cancer Cell* 2003;**3**:233-45.

16. Lee, YS, Kim, SH, Cho, JA, et al. Introduction of the CIITA gene into tumor cells produces exosomes with enhanced anti-tumor effects. *Exp Mol Med* 2011;**43**:281-90.

17. Hungermann, D, Schmidt, H, Natrajan, R, et al. Influence of whole arm loss of chromosome 16q on gene expression patterns in oestrogen receptor-positive, invasive breast cancer. *J Pathol* 2011;**224**:517-28.

18. Rath, O, Kozielski, F. Kinesins and cancer. *Nat Rev Cancer* 2012;**12**:527-39.

19. Kim, YR, Chung, NG, Kang, MR, et al. Novel somatic frameshift mutations of genes related to cell cycle and DNA damage response in gastric and colorectal cancers with microsatellite instability. *Tumori* 2010;**96**:1004-9.

20. Lyng, H, Brovig, RS, Svendsrud, DH, et al. Gene expressions and copy numbers associated with metastatic phenotypes of uterine cervical cancer. *BMC Genomics* 2006;**7**:268.

21. Yu, L, Liu, S, Guo, W, et al. Upregulation of Mad2 facilitates in vivo and in vitro osteosarcoma progression. *Oncol Rep* 2012;**28**:2170-6.

22. Yang, J, Ramnath, N, Moysich, KB, et al. Prognostic significance of MCM2, Ki-67 and gelsolin in non-small cell lung cancer. *BMC Cancer* 2006;**6**:203.

23. Nodin, B, Fridberg, M, Jonsson, L, et al. High MCM3 expression is an independent biomarker of poor prognosis and correlates with reduced RBM3 expression in a prospective cohort of malignant melanoma. *Diagn Pathol* 2012;**7**:82.

24. Liang, S, He, L, Zhao, X, et al. MicroRNA let-7f inhibits tumor invasion and metastasis by targeting MYH9 in human gastric cancer. *PLoS One* 2011;**6**:e18409.

25. Menke, V, Van Zoest, KP, Moons, LM, et al. Myo9B is associated with an increased risk of Barrett's esophagus and esophageal adenocarcinoma. *Scand J Gastroenterol* 2012;**47**:1422-8.

26. Wu, P, Walker, BA, Brewer, D, et al. A gene expression-based predictor for myeloma patients at high risk of developing bone disease on bisphosphonate treatment. *Clin Cancer Res* 2011;**17**:6347-55.

27. Whiteside, TL, Stanson, J, Shurin, MR, et al. Antigen-processing machinery in human dendritic cells: up-regulation by maturation and down-regulation by tumor cells. *J Immunol* 2004;**173**:1526-34.

28. Bric, A, Miething, C, Bialucha, CU, et al. Functional identification of tumor-suppressor genes through an in vivo RNA interference screen in a mouse lymphoma model. *Cancer Cell* 2009;**16**:324-35.

29. Feng, Y, Lee, N, Fearon, ER. TIP49 regulates beta-catenin-mediated neoplastic transformation and T-cell factor target gene induction via effects on chromatin remodeling. *Cancer Res* 2003;**63**:8726-34.

30. Osaki, H, Walf-Vorderwulbecke, V, Mangolini, M, et al. The AAA+ ATPase RUVBL2 is a critical mediator of MLL-AF9 oncogenesis. *Leukemia* 2013;**27**:1461-8.

31. Gu, Z, Li, Y, Lee, P, et al. Protein arginine methyltransferase 5 functions in opposite ways in the cytoplasm and nucleus of prostate cancer cells. *PLoS One* 2012;**7**:e44033.

32. Carvalho, L, Yu, J, Schwartsmann, G, et al. RNA expression of the molecular signature genes for metastasis in colorectal cancer. *Oncol Rep* 2011;**25**:1321-7.

33. Zhao, C, Yasui, K, Lee, CJ, et al. Elevated expression levels of NCOA3, TOP1, and TFAP2C in breast tumors as predictors of poor prognosis. *Cancer* 2003;**98**:18-23.

34. Park, JS, Kim, HS, Park, MY, et al. Topoisomerase II alpha as a universal tumor antigen: antitumor immunity in murine tumor models and H-2K(b)-restricted T cell epitope. *Cancer Immunol Immunother* 2010;**59**:747-57.

35. Haines, JW, Coster, MR, Adam, J, et al. Xrcc2 modulates spontaneous and radiation-induced tumorigenesis in Apcmin/+ mice. *Mol Cancer Res* 2010;**8**:1227-33.

36. Zhou, LP, Luan, H, Dong, XH, et al. Association between XRCC5, 6 and 7 gene polymorphisms and the risk of breast cancer: a HuGE review and meta-analysis. *Asian Pac J Cancer Prev* 2012;**13**:3637-43.

37. Fremont, S, Gerard, A, Galloux, M, et al. Beclin-1 is required for chromosome congression and proper outer kinetochore assembly. *EMBO Rep* 2013;**14**:364-72.

38. Urbanucci, A, Sahu, B, Seppala, J, et al. Overexpression of androgen receptor enhances the binding of the receptor to the chromatin in prostate cancer. *Oncogene* 2012;**31**:2153-63.

39. Alhopuro, P, Phichith, D, Tuupanen, S, et al. Unregulated smooth-muscle myosin in human intestinal neoplasia. *Proc Natl Acad Sci U S A* 2008;**105**:5513-8.

40. Hovanessian, AG, Soundaramourty, C, El Khoury, D, et al. Surface expressed nucleolin is constantly induced in tumor cells to mediate calcium-dependent ligand internalization. *PLoS One* 2010;**5**:e15787.

41. Nagathihalli, NS, Nagaraju, G. RAD51 as a potential biomarker and therapeutic target for pancreatic cancer. *Biochim Biophys Acta* 2011;**1816**:209-18.

42. Ghosh, JC, Dohi, T, Kang, BH, et al. Hsp60 regulation of tumor cell apoptosis. *J Biol Chem* 2008;**283**:5188-94.

43. Liu, Y, Carlsson, R, Ambjorn, M, et al. PD-L1 expression by neurons nearby tumors indicates better prognosis in glioblastoma patients. *J Neurosci* 2013;**33**:14231-45.

44. Natividad, KD, Junankar, SR, Mohd Redzwan, N, et al. Interleukin-27 signaling promotes immunity against endogenously arising murine tumors. *PLoS One* 2013;**8**:e57469.

45. Woo, SR, Turnis, ME, Goldberg, MV, et al. Immune inhibitory molecules LAG-3 and PD-1 synergistically regulate T-cell function to promote tumoral immune escape. *Cancer Res* 2012;**72**:917-27.

46. Jensen, SM, Maston, LD, Gough, MJ, et al. Signaling through OX40 enhances antitumor immunity. *Semin Oncol* 2010;**37**:524-32.

47. Ice, RJ, McLaughlin, SL, Livengood, RH, et al. "NEDD9 depletion destabilizes Aurora A kinase and heightens the efficacy of Aurora A inhibitors: implications for treatment of metastatic solid tumors.". *Cancer Res* 2013.

48. Montagnoli, A, Moll, J, Colotta, F. Targeting cell division cycle 7 kinase: a new approach for cancer therapy. *Clin Cancer Res* 2010;**16**:4503-8.

49. Tho, LM, Libertini, S, Rampling, R, et al. Chk1 is essential for chemical carcinogen-induced mouse skin tumorigenesis. *Oncogene* 2012;**31**:1366-75.

50. Li, G, Cai, M, Fu, D, et al. Heat shock protein 90B1 plays an oncogenic role and is a target of microRNA-223 in human osteosarcoma. *Cell Physiol Biochem* 2012;**30**:1481-90.

51. Nakamura, Y, Tanaka, F, Haraguchi, N, et al. Clinicopathological and biological significance of mitotic centromere-associated kinesin overexpression in human gastric cancer. *Br J Cancer* 2007;**97**:543-9.

52. Bernstein, SH, Venkatesh, S, Li, M, et al. The mitochondrial ATP-dependent Lon protease: a novel target in lymphoma death mediated by the synthetic triterpenoid CDDO and its derivatives. *Blood* 2012;**119**:3321-9.

53. Hu, B, Elinav, E, Huber, S, et al. Inflammation-induced tumorigenesis in the colon is regulated by caspase-1 and NLRC4. *Proc Natl Acad Sci U S A* 2010;**107**:21635-40.

54. Skotheim, RI, Autio, R, Lind, GE, et al. Novel genomic aberrations in testicular germ cell tumors by array-CGH, and associated gene expression changes. *Cell Oncol* 2006;**28**:315-26.

55. Kretschmer, C, Sterner-Kock, A, Siedentopf, F, et al. Identification of early molecular markers for breast cancer. *Mol Cancer* 2011;**10**:15.

56. Kuznetsov, SG, Haines, DC, Martin, BK, et al. Loss of Rad51c leads to embryonic lethality and modulation of Trp53-dependent tumorigenesis in mice. *Cancer Res* 2009;**69**:863-72.

57. Su, Y, Meador, JA, Calaf, GM, et al. Human RecQL4 helicase plays critical roles in prostate carcinogenesis. *Cancer Res* 2010;**70**:9207-17.

58. Partanen, JI, Tervonen, TA, Myllynen, M, et al. Tumor suppressor function of Liver kinase B1 (Lkb1) is linked to regulation of epithelial integrity. *Proc Natl Acad Sci U S A* 2012;**109**:E388-97.
