# Supplementary material for: Proton irradiation impacts age-driven modulations of cancer progression influenced by immune system transcriptome modifications from splenic tissue
Source: J Radiat Res. 2015 Aug 7;56(5):792–803. doi: 10.1093/jrr/rrv043 (PMC4577010; doi:10.1093/jrr/rrv043)
Supplement: Supplementary Data [file supp_rrv043_rrv043supp_table5.doc]

Proton Irradiated Adolescent Compared to Adolescent Non-irradiated Spleens

| **Annotation Cluster** | **Enrichment Score** | **DAVID Annotation Terms** |
| --- | --- | --- |
| 1 | 7.66 | Mitosis, nuclear division, organelle fission |
| 2 | 5.36 | Adenyl nucleotide binding, purine nucleoside binding, nucleoside binding |
| 3 | 5.15 | Antigen processing and presentation of exogenous peptide antigen via MHC class II, antigen processing and presentation of peptide antigen via MHC class II, antigen processing and presentation of peptide or polysaccharide antigen via MHC class II |
| 4 | 3.70 | Class II histocompatibility antigen, MHC class II protein complex, MHC II |
| 5 | 3.60 | RNA transport, establishment of RNA localization, nucleic acid transport, nucleobase (nucleoside) nucleotide and nucleic acid transport |
| 6 | 3.41 | IGc1, immunoglobulin C1-set, immunoglobulin/major histocompatibility complex (conserved site) |
| 7 | 3.15 | Sm, like-Sm ribonucleoprotein eukaryotic and archaea-type (core), like-Sm ribonucleoprotein (core) |
| 8 | 3.09 | Proteasome beta-type subunit (conserved site), proteasome alpha and beta subunits, proteasome subunit alpha/beta, proteasome core complex, threonine-type peptidase activity, threonine-type endopeptidase activity, threonine protease |
| 9 | 2.63 | MHC protein complex, Graft-versus-host disease, type 1 diabetes mellitus |
| 10 | 2.25 | Stress fiber, actin filament bundle, actomyosin |

**Supplemental Table 5.** The top 10 functional annotation clusters determined from key genes for proton irradiated adolescent and non-irradiated adolescent spleens compared to all other groups. This was determined through DAVID Gene Functional Classification Tool. The enrichment scores were determined by DAVID through the geometric mean of the EASE scores (modified Fisher Exact).
