# Supplementary material for: Proton irradiation impacts age-driven modulations of cancer progression influenced by immune system transcriptome modifications from splenic tissue
Source: J Radiat Res. 2015 Aug 7;56(5):792–803. doi: 10.1093/jrr/rrv043 (PMC4577010; doi:10.1093/jrr/rrv043)
Supplement: Supplementary Data [file supp_rrv043_rrv043supp_table6.doc]

Proton Irradiated Old Compared to Proton Irradiated Adolescent Spleens

| **Annotation Cluster** | **Enrichment Score** | **DAVID Annotation Terms** |
| --- | --- | --- |
| 1 | 10.27 | Adenyl nucleotide binding, purine nucleoside binding, nucleoside binding |
| 2 | 7.62 | Mitosis, nuclear division, organelle fission |
| 3 | 6.27 | IGc1, immunoglobulin C1-set, immunoglobulin/major histocompatibility complex (conserved site) |
| 4 | 4.93 | Establishment of RNA localization, nucleic acid transport, RNA transport, RNA localization, nucleobase (nucleoside) nucleotide and nucleic acid transport |
| 5 | 4.75 | MHC class II protein complex, class II histocompatibility antigen, MHC II |
| 6 | 2.94 | Sm, like-Sm ribonucleoprotein eukaryotic and archaea-type core, like-Sm ribonucleoprotein (core) |
| 7 | 2.90 | Proteasome beta-type subunit (conserved site), proteasome alpha and beta subunits, proteasome subunit alpha/beta, proteasome core complex, threonine-type endopeptidase activity, threonine-type peptidase activity, threonine protease |
| 8 | 2.72 | MCM, DNA-dependent ATPase MCM, DNA-dependent ATPase MCM (conserved site) |
| 9 | 2.18 | Positive regulation of T cell mediated cytotoxicity, regulation of T cell mediated cytotoxicity, positive regulation of T cell mediated immunity |
| 10 | 1.97 | DNA unwinding during replication, DNA duplex unwinding, DNA geometric change |

**Supplemental Table 6.** The top 10 functional annotation clusters determined from key genes for proton irradiated old and proton irradiated adolescent spleens compared to all other groups. This was determined through DAVID Gene Functional Classification Tool. The enrichment scores were determined by DAVID through the geometric mean of the EASE scores (modified Fisher Exact).
