# Supplementary material for: Proton irradiation impacts age-driven modulations of cancer progression influenced by immune system transcriptome modifications from splenic tissue
Source: J Radiat Res. 2015 Aug 7;56(5):792–803. doi: 10.1093/jrr/rrv043 (PMC4577010; doi:10.1093/jrr/rrv043)
Supplement: Supplementary Data [file supp_rrv043_rrv043supp_table7.doc]

Proton Irradiated Old Compared to Old Non-irradiated Spleens

| **Annotation Cluster** | **Enrichment Score** | **DAVID Annotation Terms** |
| --- | --- | --- |
| 1 | 6.01 | Imuunoglobulin C1-set, IGc1, allograft rejection, immunologic conserved site, autoimmune thyroid disease |
| 2 | 5.58 | Antigen processing and presentation of exogenous peptide antigen via MHC class II, antigen processing and presentation of peptide antigen via MHC class II, antigen processing and presentation of peptide or polysaccharide antigen via MHC class II |
| 3 | 3.50 | MHC class II alpha/beta chain N-terminal, MHC class II protein complex, class II histocompatibility antigen, MHC II |
| 4 | 3.28 | RNA transport, nucleic acid transport, establishment of RNA localization, RNA localization, nucleobase (nucleoside) nucleotide and nucleic acid transport |
| 5 | 3.06 | Class I histocompatibility antigen, MHC class I alpha chain (alpha 1 &2), MHC class I protein complex, MHC class 1-like antigen recognition |
| 6 | 2.96 | Adenyl nucleotide binding, purine nucleoside binding, nucleoside binding |
| 7 | 2.86 | Mitosis, nuclear division, M phase of mitotic cell cycle, organelle fission |
| 8 | 2.44 | Positive regulation of cell killing, positive regulation of leukocyte mediated cytotoxicity, regulation of leukocyte mediated cytotoxicity, regulation of cell killing |
| 9 | 1.97 | RNA recognition motif RNP-1, nucleotide-binding (alpha-beta plait), RRM |
| 10 | 1.96 | Antigen processing and presentation of peptide antigen via MHC class I, MHC I |

**Supplemental Table 7.** The top 10 functional annotation clusters determined from key genes for proton irradiated old and non-irradiated old spleens compared to all other groups. This was determined through DAVID Gene Functional Classification Tool. The enrichment scores were determined by DAVID through the geometric mean of the EASE scores (modified Fisher Exact).
