# Supplementary material for: Proton irradiation impacts age-driven modulations of cancer progression influenced by immune system transcriptome modifications from splenic tissue
Source: J Radiat Res. 2015 Aug 7;56(5):792–803. doi: 10.1093/jrr/rrv043 (PMC4577010; doi:10.1093/jrr/rrv043)
Supplement: Supplementary Data [file supp_rrv043_rrv043supp_table8.doc]

Non-Irradiated Old Compared to Adolescent Non-irradiated Spleens

| **Annotation Cluster** | **Enrichment Score** | **DAVID Annotation Terms** |
| --- | --- | --- |
| 1 | 14.98 | Immunoglobulin C1-set, IGc1, immunoglobulin/major histocompatibility complex (conserved site) |
| 2 | 8.77 | Antigen processing and presentation of exogenous peptide antigen via MHC class II, antigen processing and presentation of peptide antigen via MHC class II, antigen processing and presentation of peptide or polysaccharide antigen via MHC class II |
| 3 | 7.05 | MHC class II protein complex, MHC II, class II histocompatibility antigen |
| 4 | 5.79 | Class I histocompatibility antigen, MHC class I alpha chain (alpha 1 and alpha 2), MHC class I-like antigen recognition |
| 5 | 4.43 | Regulation of lymphocyte activation, regulation of leukocyte activation, regulation of cell activation |
| 6 | 3.75 | Positive regulation of leukocyte mediated immunity, positive regulation of lymphocyte mediated immunity, positive regulation of immune effector process |
| 7 | 3.62 | positive regulation of adaptive immune response based on somatic recombination of immune receptors built from immunoglobulin superfamily domains, positive regulation of adaptive immune response, regulation of adaptive immune response based on somatic recombination of immune receptors built from immunoglobulin superfamily domains, regulation of adaptive immune response |
| 8 | 3.36 | Positive regulation of leukocyte mediated cytotoxicity, positive regulation of cell killing, regulation of leukocyte mediated cytotoxicity, regulation of cell killing |
| 9 | 3.33 | Immunoglobulin mediated immune response, B cell mediated immunity, lymphocyte mediated immunity, adaptive immune response based on somatic recombination of immune receptors built from immunoglobulin superfamily domains, adaptive immune response |
| 10 | 3.24 | Positive regulation of T cell mediated cytotoxicity, regulation of T cell mediated cytotoxicity, positive regulation of T cell mediated immunity, regulation of T cell mediated immunity |

**Supplemental Table 8.** The top 10 functional annotation clusters determined from key genes for non-irradiated old and non-irradiated adolescent spleens compared to all other groups. This was determined through DAVID Gene Functional Classification Tool. The enrichment scores were determined by DAVID through the geometric mean of the EASE scores (modified Fisher Exact).
